# COVID-19 and the gastrointestinal tract: what do we already know?

**DOI:** 10.31744/einstein_journal/2020RW5909

**Published:** 2020-11-05

**Authors:** Joana Ferro Machado de Almeida, Ethel Zimberg Chehter

**Affiliations:** 1 Faculdade de Medicina do ABC Santo AndréSP Brazil Faculdade de Medicina do ABC, Santo André, SP, Brazil.

**Keywords:** SARS-CoV-2, Betacoronavirus, Coronavirus infections, COVID-19, Gastrointestinal tract, Gastrointestinal diseases/etiology

## Abstract

The new coronavirus disease pandemic is defining 2020, with almost 17.5 million infected individuals and 700 thousand deaths up to beginning of August. It is caused by SARS-CoV-2 and the transmission is through the respiratory tract. Those infected may be asymptomatic, present typical symptoms (fever, dry cough and dyspnea), gastrointestinal symptoms (diarrhea, nausea, vomiting and abdominal pain) and viral RNA in stools. The objective of this work was to review the literature related to the prevalence of gastrointestinal symptoms, and to check the possibility of fecal-oral transmission. We searched PubMed^®^ database on COVID-19 and gastrointestinal tract and selected articles using the PRISMA method. We eliminated articles based on titles and abstracts, small number of patients and the mechanism of infection, leaving 14 studies. Comorbidities and laboratory alterations (elevation of hepatic aminotransferases and bilirubin) were related to worsening of the disease. The prevalence of gastrointestinal symptoms ranged from 6.8% to 61.3%, including diarrhea (8.14% to 33.7%), nausea/vomiting (1.53% to 26.4%), anorexia (12.1% to 40.0%) and abdominal pain (0% to 14.5%). The presence of viral RNA in stools was rarely tested, but positive in 0% to 48.1%. The gastrointestinal tract is affected by COVID-19, causing specific symptoms, laboratory alterations and viral presence in the feces. However, the results of prevalence and possibility of fecal-oral transmission were varied, requiring further studies for more assertive conclusions. It is important that healthcare professionals draw attention to this fact, since these changes can help make diagnosis and initiate early treatment.

## INTRODUCTION

In December 2019, the first cases of patients infected with the new coronavirus were identified in Wuhan, China.^(^^1^^)^ The year 2020 will be remembered by the 2019 coronavirus pandemic (COVID-19), causing a great impact on public health and economy of many countries. On August 1^st^, 2020, according to the World Health Organization (WHO) website, there were 17,396,943 cumulative cases and 675,060 deaths worldwide. The country with the highest number of infected individuals and deaths is the United States, with 4,456,389 and 151,265, respectively. In Brazil, these figures were 2,610,102 cases and 91,263 deaths.^(^^2^^)^

The virus, called severe acute respiratory syndrome coronavirus 2 (SARS-CoV-2), is the seventh coronavirus known to infect humans. The others are the acute severe respiratory syndrome coronavirus (SARS-CoV), with an epidemic in 2003, and the Middle Eastern respiratory syndrome coronavirus (MERS-CoV), with an epidemic in the Middle East, in 2012, and both cause severe disease; and HKU1, NL63, OC43, and 229E, which cause mild symptoms. SARS-CoV-2 is an encapsulated RNA virus of the order *Nidovirales*, family *Coronaviridae*, subfamily *Coronavirinae*, genus Beta.^(^^3^^)^ SARS-CoV-2 probably originated in bats and is transmitted to humans through another animal.^(^^4^^)^ The main transmission route is respiratory, through contact with droplets, aerosols, and contaminated surfaces.^(^^5^^)^

Typical symptoms of the disease are fever, dry cough, dyspnea, headache, anosmia, dysgeusia, and pneumonia, but it can also be asymptomatic. It may cause progressive respiratory failure through alveolar injury, which may lead to death.^(^^1^^,^^6^^)^ However, several studies indicate that patients may present symptoms related to the gastrointestinal tract (GIT), with the presence of the virus in epithelial cells of the GIT and its RNA in feces. Based on this, a possible oral-fecal transmission of the disease is suggested.^(^^6^^,^^7^^)^ The main gastrointestinal symptoms are diarrhea, nausea, vomiting, and abdominal pain.^(^^8^^)^

The SARS-CoV-2 virus uses the angiotensin 2 converting enzyme (ACE2) as a receptor for entrance into cells. It mainly infects lung cells, but may have gastrointestinal involvement, which also has ACE2 receptors at the brush border of the intestinal mucosa.^(^^6^^)^ In addition, the virus is released at the apical part of the lung cells. Dragged by mucociliary movement, it can gain the GIT. These ACE2 receptors operate diminishing the action of the renin-angiotensin-aldosterone system by metabolizing angiotensin 2. At high serum levels, angiotensin 2 has effects on cardiovascular, renal, and respiratory systems, and may cause chronic hypertension and renal and respiratory failure. The SARS-CoV-2 virus causes downregulation and blockage of these ACE2 receptors, which explains the clinical picture of pulmonary failure and adult respiratory distress syndrome in some infected patients.^(^^9^^)^

The viral surface has S (Spike) glycoproteins, which need to be separated into S1 and S2 domains, so that the virus can adhere to the cell membrane. Furin and transmembrane serine protease (TMPRSS2) are substances that allow this separation. Thus, S1 binds to the ACE2 receptor and S2 to the cell membrane, allowing the virus to enter the cell through endocytosis. Furin and TMPRSS2 are enzymes present in the cells of the small intestine; the former also acts in the activation of toxins of some microorganisms.^(^^7^^,^^9^^)^

ACE2 of the intestinal mucosa is associated with the amino acid carrier, B^0^AT1, and regulates the intestinal flora. This occurs because this transporter allows the absorption of tryptophan, which stimulates the mTOR path to produce antimicrobial peptides. Thus, the SARS-CoV-2 infection changes the amount, and blocks the ACE2 receptors in the brush edge, causing tryptophan deficiency and lower production of antimicrobial peptides, which in turn can cause changes in the intestinal microbioma and inflammation.^(^^7^^,^^9^^)^

Patients with COVID-19 present with a great inflammatory reaction caused by the so-called cytokine storm, which can be originated or potentiated by the GIT. The small intestine has the largest amount of lymphoid tissue in the body, with Peyer patches, mesenteric lymph nodes, and lymphoid follicles along the intestine. In the mucosa and below the lamina propria, the intestines also have a large population of activated T cells, plasma cells, mast cells, dendritic cells, and macrophages. In the scenario of an infection such as COVID-19, there is an exaggerated release of cytokines, which promote the recruitment of several other cells, causing a great inflammatory process.^(^^9^^,^^10^^)^ Studies point out that interferon and influenza virus infection (interferon pathway inducer) may be related to increased ACE2 transcription.^(^^11^^)^ Smokers and patients with chronic obstructive pulmonary disease (COPD) also have a higher expression of ACE2 receptors.^(^^12^^)^ In addition, diabetes and hypertension have been associated with polymorphisms of the ACE2 gene.^(^^7^^)^

Drugs used in the treatment of hypertension, such as ACE inhibitors (ACEi) and angiotensin receptor blockers (ARB), reduce inflammation by decreasing cytokines. In addition, they may increase the expression of ACE2.^(^^13^^)^ However, the use of ACEi and ARB has not been associated with a worse clinical evolution in patients with COVID-19. Thus, maintenance of the use of these drugs in hypertensive patients is recommended.^(^^14^^,^^15^^)^

While the SARS-CoV and SARS-CoV-2 viruses use ACE2 as a mediator to enter the cells, MERS-CoV uses dipeptidyl-peptidase 4 (DPP4).^(^^7^^)^ Because they are viruses from the same family, some authors have studied whether DPP4 is also involved in the infection mechanism of the new disease. According to Vankadari et al., the S1 domain of the SARS-CoV-2 membrane glycoprotein, besides interacting with ACE2, the main gateway for the new virus may have some relation with DPP4.^(^^16^^)^ However, the study by Tai et al., found that the virus did not bind to 293T cells that expressed only DPP4. This connection only occurred in 293T cells with ACE2.^(^^17^^)^

Several articles on GIT-related symptoms of COVID-19 and its possible oral-fecal transmission have been published, with divergent results.

The objective of this study was to review the literature related to the prevalence of gastrointestinal symptoms, and to verify the possibility of fecal-oral transmission of the disease.

## METHODS

**Figure 1:**
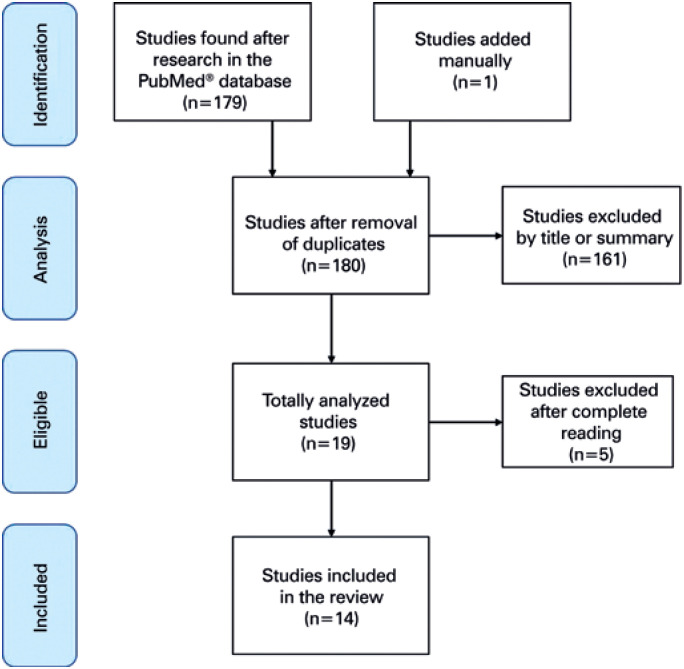
Preferred Reporting Items for Systematic Reviews and Meta-Analyses Diagram (PRISMA)

The database chosen for this work was PubMed^®^. On May 5, 2020, we searched for articles with the words “COVID-19” or “SARS-CoV-2” and “GIT” or “gastrointestinal” or “enteric.” A total of 179 articles resulted from this search. The inclusion criteria for the studies in this review were the fulfillment of eligibility criteria (articles addressing SARS-CoV-2 infection and GIT symptoms), articles in English, and samples with more than 50 patients. Exclusion criteria were study design of case reports, reviews on the infection mechanism and virus, GIT-related procedure recommendation manuals in infected patients, articles in languages other than English, and less than 50 patients evaluated. Figure 1 shows the diagram Preferred Reporting Items for Systematic Reviews and Meta-Analyses (PRISMA) performed based on reading titles and abstracts; 161 articles were eliminated, leaving 18 articles. One last article was added later. Of these 19 articles, four were reviews about the virus and its mechanism of infection, which were disregarded. Finally, a study including only ten patients was eliminated. With this, the review was carried out with 14 articles, in that four were meta-analyses, one systematic review, and nine retrospective studies. The analysis of the articles was initially made by the author, but the final choice also included a senior reviewer. Due to the small number of large studies related to this subject, and the importance and urgency of the topic, several variations of study designs were included. Information on country of origin, mean age, different comorbidities, typical symptoms (fever, cough, and dyspnea, among others), gastrointestinal symptoms (diarrhea, nausea, vomiting, and abdominal pain), and the presence of viral RNA in feces, when cited, were included in this study for analysis.

## RESULTS

As to retrospective studies, three were done only with patients from the United States and six only from China. The systematic review included several studies carried out in China. As to the meta-analyses, two of them included studies done only in China and two evaluated various countries. Tables 1 to 3 present general data, typical symptoms, and gastrointestinal symptoms of patients with COVID-19 of the retrospective studies, respectively. Table 4 displays the symptoms of patients with the disease in the meta-analyses.[Table t2]

**Table 1:** General data of patients with COVID-19 of the nine retrospective studies

	Jin et al.^(^^18^^)^			Lin et al.^(^^19^^)^				Pan et al.^(^^20^^)^			
	With GI symptoms	Without GI symptoms	p value	Total	With GI symptoms	Without GI symptoms	p value	Total	With GI symptoms	Without GI symptoms	p value
Patients	74	577		95	58	37		204	103	101	
Age	46.14±14.19	45.09±14.45	0.559	43.3±18.3	48.0±17.1	41.1±19.5	0.073	52.91±15.98	52.21±15.92	53.61±16.10	0.533
Sex	M: 37/74 (50.0) W: 37/74 (50.0)	M: 294/577 (51) W: 283/577 (49)	M: 0.902	M: 45/95 (47.4) W: 50/95 (52.6)	M: 27/58 (46.6) W: 31/58 (53.4)	M: 18/37 (48.6) W: 19/37 (51.4)	W: 0.84	M: 107/204 (52.5) W: 97/204 (47.5)	M: 55/103 (53.4) W: 48/103 (46.6)	M: 52/101 (51.5) W: 49/101 (48.5)	0.784
BMI, kg/m^2^											
Tobacco use	3/74 (4.23)	38/577 (6.59)	0.610	6/95 (6.3)	5/58 (8.6)	1/37 (2.7)	0.40				
Alcohol use				9/95 (9.5)	6/58 (10.3)	3/37 (8.1)	1.00				
Any comorbidity	25/74 (33.78)	153/577 (26.52)	0.212								
Coronary artery disease											
Congestive heart failure											
Arrhythmia											
Hypertension	12/74 (16.22)	88/577 (15.25)	0.864	16/95 (16.8)	10/58 (17.2)	6/37 (16.2)	0.90				
*Diabetes mellitus*	7/74 (9.46)	41/577 (7.11)	0.477	6/95 (6.3)	3/58 (5.2)	3/27 (8.1)	0.67				
Cerebrovascular disease				4/95 (4.2)	3/58 (5.2)	1/37 (2.7)	1.00				
Pulmonary disease	0/74 (0)	1/577 (0.17)	1.00	5/95 (5.3)	1/58 (1.7)	4/37 (10.8)	0.074	9/204 (4.41)	7/103 (6.80)	2/101 (1.98)	0.182
Chronic renal disease	0/74 (0)	6/577 (1.04)	1.00	1/95 (1.1)	1/58 (1.7)	0/37 (0)	1.00				
Chronic liver disease	8/74 (10.81)	17/577 (2.95)	0.004					1/204 (0.97)			
Cancer	0/74 (0)	6/577 (1.04)	1.00	5/95 (5.3)	4/58 (6.9)	1/37 (2.7)	0.65	13/204 (6.37)	8/103 (7.77)	5/101 (4.95)	0.410
Cardiovascular disease	1/74 (1.35)	4/577 (0.69)	0.454					44/204 (21.57)	23/103 (22.3)	21/101 (20.79)	0.789
Immunossuppression	0/74 (0)	1/577 (0.17)	1.00								
Viral RNA in feces	3/9 (33.33)	0/577 (0)		31/65 (47.7)	22/42 (52.4)	9/23 (39.1)	0.31				
Oxygen supplementation											
ICU	5/74 (6.76)	12/577 (2.08)	0.034					16/204 (7.84)	6/103 (5.94)	10/101 (9.90)	0.279
Mechanical ventilation	5/74 (6.76)	12/577 (2.08)	0.034								
Death				0/95 (0)	0/58 (0)	0/37 (0)		36/204 (17.65)	19/103 (18.45)	17/101 (16.83)	0.762

	Wan et al.^(^^21^^)^				Redd et al.^(^^22^^)^				Nobel et al.^(^^23^^)^	Cholankeril et al.^(^^24^^)^
	Total	With GI symptoms	Without GI symptoms	p value	Total	With GI symptoms	Without GI symptoms	p value	Total	Total
Patients	230	49	181		318	195	123		278	116
Age	47.5 (36.8-60)	55 (40-65)	46 (36-57)	0.017	63.4±16.6	62.3±15.9	65.0±17.6	0.16	18-30: 31/278 (11)	50 (35-67)
									31-50: 69/278 (25)	
									51-70: 103/278 (37)	
									>70: 75/278 (27)	
Sex	M: 129/230 (56) W: 101/230 (44)	M: 27/49 (55) W: 22/49 (45)	M: 102/181 (56) W: 79/181 (22)	0.87	M: 174/318 (54.7) W: 144/318 (45.3)	M: 102/195 (52.3) W: 93/195 (47.7)	M: 72/123 (58.6) W: 51/123 (35.4)	W: 0.28	M: 145/278 (52) W: 133/278 (48)	M: 62/116 (53.4) W: 54/116 (46.6)
BMI. kg/m^2^					30.0±6.5	30.5±6.7	29.3±6.2	0.11	<25.0: 25/278 (9)	25.8 (23.2-31.5)
									25.0-29.9: 43/278 (16)	
									≥30.0: 47/278 (17)	
									Without evaluation: 165/278 (59)	
Tobacco use					35/318 (11.0)	23/195 (11.8)	12/123 (9.8)	0.57		Former smoker: 21 (23.3) Smoker: 3 (2.8)
Alcohol use					39/318 (12.3)	28/195 (14.4)	11/123 (8.9)	0.15		
Any comorbidity	58/230 (25)	19/49 (39)	39/181 (22)	0.017						
Coronary disease					46/318 (14.5)	26/195 (13.3)	20/123 (16.3)			
Congestive heart failure					31/318 (9.8)	16/195 (8.3)	15/123 (12.2)	0.25		
Arrhythmia					48/318 (15.1)	26/195 (13.3)	22/123 (17.9)	0.27		
Hypertension	40 (17)				188/318 (59.1)	111/195 (56.9)	77/123 (62.6)	0.32		32 (27.8)
*Diabetes mellitus*	11 (5)				105/318 (33.1)	62/195 (32.0)	43/123 (35.0)	0.58		19 (16.4)
Cerebrovascular disease					11/318 (3.5)	8/195 (4.1)	3/123 (2.4)	0.42		
Pulmonary disease (%)	7 (3)				67/318 (21.1)	40/195 (20.5)	27/123 (22.0)	0.76		24 (20.7)
Chronic renal disease					40/318 (13.8)	20/195 (11.7)	20/123 (17.0)	0.20		4 (3.5)
Chronic hepatic disease	6 (3)									3 (2.8)
Cancer	3 (1)									
Cardiovascular disease	10 (4)									15 (12.9)
Immunosuppressant										4 (4.3)
Viral RNA in feces										
Oxygen supplementation		44/49 (90)	143/181 (79)	0.10						
ICU		15/49 (31)	20/181 (11)	0.0015	35/202 (17.5)	20 (15.4)	15 (21.4)	0.28	44/278 (16)	
Mechanical ventilation		6/49 (12)	3/181 (2)	0.0036	26/202 (13.0)	14 (10.9)	12 (16.0)	0.22		
Death		4/49 (8)	2/181 (1)	0.020	32/202 (15.8)	16 (12.2)	16 (22.5)	0.06	9/278 (3.2)	
	**Zhang et al.**^(^^25^^)^	**Zhou et al.**^(^^26^^)^								
	**Total**	**Total**	**Total medical team**	**With GI symptoms - medical team**	**No GI symptoms - medical team**	**p value**	**Total non-medical**	**With GI symptoms - non-medical**	**Without GI symptoms - non-medical**	**p value**
Patients	140	254	93	23	70		161	43	118	
Age	57 (25-87)	50 (36-65)	36 (31-41)	35 (30-40)	36 (31-42)	0.614	62 (49-69)	61 (49-67)	62 (49-70)	0.615
Sex	M: 71/140 (50.7) W: 69/140 (49.3)	M: 115/254 (45.3) W: 139/254 (54.7)	M: 32/93 (34) W: 61/93 (66)	M: 6/23 (26) W:17/23 (74)	M: 26/70 (37) W:44/70 (63)	0.45	M: 83/161 (52) W: 78/161 (48)	M: 16/43 (37) W: 27/43 (63)	M: 67/118 (57) W: 51/118 (43)	0.033
BMI, kg/m^2^											
Tobacco use	Former smoker: 7/140 (5.0) Smoker: 2/140 (1.4)									
Alcohol use											
Any comorbidity	90/140 (64.3)									
Coronary disease	7/140 (5.0)	17/254 (6.7)	2/93 (2.1)	0/23 (0)	2/70 (3)	1.00	15/161 (9)	6/43 (14)	9/118 (8)	0.231
Congestive heart failure											
Arrhythmia	5/140 (3.6)									
Hypertension	42/140 (30.0)	63/254 (24.8)	6/93 (6.4)	0/23 (0)	6/70 (9)	0.33	57/161 (35)	14/43 (33)	43/118 (36)	0.712
*Diabetes mellitus*	17/140 (12.1)	26/254 (10.2)	3/93 (3.2)	0/23 (0)	3/70 (4)	0.572	23/161 (14)	4/43 (9)	19/118 (16)	0.321
Cerebrovascular disease	3/140 (2.1)	13/254 (5.1)	1/93 (1.1)	0/23 (0)	1/70 (1)	1.00	12/161 (7)	3/43 (7)	9/118 (8)	1.00
Pulmonary disease	2/140 (1.4)	6/254 (2.4)	1/93 (1.1)	0/23 (0)	1/70 (1)	1.00	5/161 (3)	2/43 (5)	3/118 (3)	0.61
Chronic renal disease	2/140 (1.4)									
Chronic liver disease		3/254 (1.2)	1/93 (1.1)	0/23 (0)	1/70 (1)	1.00	2/161 (1)	0/43 (0)	2/118 (2)	1.00
Cancer		2/254 (0.8)	1/93 (1.1)	0/23 (0)	1/70 (1)	1.00	1/161 (1)	0/43 (0)	1/118 (1)	1.00
Cardiovascular disease											
Immunosuppression		1/254 (0.4)	0/93 (0)	0/23 (0)	0/70 (0)		1/161 (1)	1/43 (2)	0/118 (0)	0.267
Viral RNA in feces											
Oxygen supplementation											
ICU											
Mechanical ventilation		18/254 (7)	5/93 (5.4)	1/23 (4.3)	4/70 (5.7)	1.00	13/161 (8)	2/43 (4.7)	11/118 (9.3)	0.516
Death		16/254 (6.3)	2/93 (2.1)	1/23 (4.3)	1/70 (1.43)	0.435	14/161 (9)	3/43 (7)	11/118 (9.3)	0.457

Results expressed as n. mean±standard deviation. n/n total (%).

GI: gastrointestinal; M: men; W: women; BMI: body mass index; ICU: intensive care unit.

**Table 2 t2:** Typical symptoms of patients with COVID-19 in the nine retrospective studies

Symptoms	Jin et al.^(^^18^^)^			Lin et al.^(^^19^^)^	Pan et al.^(^^20^^)^			Wan et al.^(^^21^^)^	Redd et al.^(^^22^^)^			
	With GI symptoms	Without GI symptoms	p value	Total	Total	With GI symptoms	Without GI symptoms	Total	Total	With GI symptoms	Without GI symptoms	p value
Patients	74	577		95	204	103	101	230	318	195	123	
Fever	63/74 (85.14) >38.5°C: 29/74 (39.19)	482/577 (83.54) >38.5°C: 101/577 (17.50)	0.867 >38.5°C: <0.001	Yes		95/103 (92.23)		193/230 (84)	258/318 (81.3)	161/195 (82.6)	97/123 (78.9)	0.41
Fatigue	23/74 (31.08)	96/577 (16.64)	0.004			54/103 (52.42)		43 (19)	183/318 (57.5)	127/195 (65.1)	56/123 (45.5)	0.0006
Myalgia	10/74 (13.51)	61/577 (10.57)	0.430			15/103 (14.56)			123/318 (38.7)	96/195 (49.2)	27/123 (22.0)	<0.0001
Chills									72/318 (22.6)	50/195 (25.6)	22/123 (17.9)	0.11
Diaphoresis									15/318 (4.7)	12/195 (6.2)	3/123 (2.4)	0.13
Arthralgia									8/318 (2.5)	4/195 (2.1)	4/123 (3.3)	0.51
Dry cough	53/74 (71.62)	382/577 (66.20)	0.431					159/230 (69)	247/318 (77.7)	156/195 (80.0)	91/123 (74.0)	0.21
Productive cough	29/74 (39.19)	198/577 (34.32)	0.438					98/230 (43)	45/318 (14.2)	33/195 (16.9)	12/123 (9.8)	0.74
Dyspnea	8/74 (10.81)	19/577 (3.30)	0.007					30 (13)	191/318 (60.1)	107/195 (54.9)	84/123 (68.3)	0.02
Sore throat	6/74 (8.11)	93/577 (16.12)	0.085						54/318 (17.0)	42/195 (21.5)	12/123 (9.8)	0.0064
Rhinorrhea									36/318 (11.4)	26/195 (13.4)	10/123 (8.1)	0.15
Nasal obstruction	2/74 (2.70)	35/577 (6.07)	0.419									
Dizziness												
Anosmia									32/318 (10.1)	26/195 (13.3)	6/123 (4.9)	0.0146
Ageusia									24/318 (7.6)	21/195 (10.9)	3/123 (2.4)	0.0057
Headache	16/74 (21.62)	51/577 (8.84)	0.002					19 (8)				
Hemoptysis	3/74 (4.05)	8/577 (1.39)	0.119					3 (1)				
**Symptoms**	**Nobel et al.**^(^^23^^)^	**Cholankeril et al.**^(^^24^^)^	**Zhang et al.**^(^^25^^)^	**Zhou et al.**^(^^26^^)^								
	**Total**	**Total**	**Total**	**Total**	**Total - Medical team**	**With GI symptoms - medical team**	**Without GI symptoms - medical team**	**p value**	**Total non-medical**	**With GI symptoms - non-medical**	**Without GI symptoms - non-medical**	**p value**
Patients	278	116	140	254	93	23	70		161	43	118	
Fever		76.70	110/120 (91.7)	213/254 (83.9)	80/93 (86)	19/23 (83)	61/70 (87)	0.729	133/161 (82)	39/43 (91)	94/118 (80)	0.157
Fatigue			90/120 (75.0)	133/254 (52.4)	52/93 (56.)	12/23 (52)	40/70 (57)	0.809	81/161 (50)	29/43 (67)	52/118 (44)	0.012
Myalgia		52.50		86/254 (33.9)	41/93 (44.)	10/23 (44)	31/70 (44)	1	45/161 (28)	17/43 (40)	28/118 (24)	0.073
Chills												
Diaphoresis												
Arthralgia												
Dry cough		94.8	90/120 (75.0)	98/254 (38.6)	41/93 (44)	7/23 (30)	34/70 (49)	0.152	57/161 (35)	14/43 (33)	43/118 (36)	0.712
Productive cough				107/254 (42.1)	31/93 (33)	6/23 (26)	25/70 (36)	0.454	76/161 (47)	17/43 (40)	59/118 (50)	0.286
Dyspnea		58	44/120 (36.7)	10/254 (3.9)	2/93 (2.1)	1/23 (4)	1/70 (1)	0.435	8/161 (5.0)	2/43 (5)	6/118 (5)	1.00
Sore throat				16/254 (6.3)	6/93 (6.4)	0/23 (0)	6/70 (9)	0.33	10/161 (6.2)	6/43 (14)	4/118 (3)	0.023
Rhinorrhea												
Nasal obstruction												
Dizziness				18/254 (7.1)	10/93 (11)	4/23 (17)	6/70 (9)	0.256	8/161 (5.0)	5/43 (12)	3/118 (3)	0.032
Anosmia												
Ageusia												
Headache												
Hemoptysis												

Results expressed as n, mean±standard deviation, n/n total (%).

GI: gastrointestinal.

**Table 3:** Gastrointestinal symptoms of patients with COVID-19 in the nine retrospective studies

	Jin et al.^(^^18^^)^	Lin et al.^(^^19^^)^			Pan L et al.^(^^20^^)^	Wan et al.^(^^21^^)^	Redd et al.^(^^22^^)^	Nobel et al.^(^^23^^)^	Cholankeril et al.^(^^24^^)^	Zhang et al.^(^^25^^)^	Zhou et al.^(^^26^^)^		
		Total	At admission	During hospitalization							Total	With GI symptoms - medical team	With GI symptoms - GI non-medical
Patients	651	95			204	230	318	278	116	140	254	23	43
GIT symptoms	74/651 (11.4)	58/95 (61.1)	11/95 (11.6)	47/95 (49.5)	103/204 (50.5)	49/230 (21)	195/318 (61.3)	97/278 (35)	37 (31.9)	55/139 (39.6)	66/254 (26.0)	23/23 (100)	43/43 (100)
Diarrhea	53/651 (8.14)	23/95 (24.2)	5/95 (5.3)	18/95 (18.9)	35/103 (33.98)	49/230 (21)	107/318 (33.7)	56/278 (20)	12 (10.3)	18/139 (12.9)	46/254 (18.1)	19/23 (86.6)	27/43 (62.8)
N/V	N: 10/651 (1.53)	N: 17/95 (17.9)	N: 3/95 (3.2)	N: 14/95 (14.7)	V: 4/103 (3.88)		N: 84/318 (26.4)	63/278 (23)	12 (10.3)	N: 24/139 (17.3)	N: 21/254 (8.3)	N: 5/23 (21.7)	N: 16/43 (37.2)
	V: 11/651 (1.69)	V: 4/95 (4.2)	V: 0/95 (0)	V: 4/95 (4.2)			V: 49/318 (15.4)			V: 7/139 (5.0)	V: 15/254 (5.9)	V: 1/23 (4.3)	V: 14/43 (32.6)
Anorexia		17/95 (17.9)	5/95 (5.3)	12/95 (12.6)	81/103 (78.64)		110/318 (34.8)		22 (25.3)	17/139 (12.1)			
Abdominal pain		2/95 (2.1)	0/95 (0)	2/95 (2.1)	2/103 (1.94)	3/230 (1)	46/318 (14.5)		10 (8.8)	8/139 (5.8)	3/254 (1.2)	0/23 (0)	3/43 (7.0)
Weight loss							30/318 (9.4)						
Melena						10/230 (4)	2/318 (0.63)						
Reflux		2/95 (2.1)	1/95 (1.1)	1/95 (1.1)			2/318 (0.63)						
Dysphagia							1/318 (0.31)						
Odynophagia							1/318 (0.31)						
Hematochezia/UGIB		2/95 (2.1)	0/95 (0)	2/95 (2.1)			1/318 (0.31)						
Constipation							3/318 (0.94)						

Results expressed as n or n/n total (%).

GI: gastrointestinal; GIT: gastrointestinal tract; N: nausea; V: vomiting; UGIB: upper gastrointestinal bleeding.

**Table 4:** Typical and gastrointestinal symptoms of patients with COVID-19 in the meta-analyses

	Sultan et al.^(^^8^^)^	Cheung et al.^(^^27^^)^ meta-analysis	Cheung et al.^(^^27^^)^ Hong Kong cohort	Cao et al.^(^^28^^)^	Li et al.^(^^29^^)^
Patients	10,890	4,243	59	46,959	1,994
Age		45.1 (IQR: 41.0-54.8)	58.5 (IQR: 43.5-68.0; range: 22-96)	46.62 (95%CI: 31.710-61.531)	
Sex		M: 57.3	M: 27/59 (45.8)	M: 55.6 (95%CI: 0.530-0.602)	M: 60 (95%CI: 0.54-0.65)
Comorbidities				35.6 (95%CI: 0.267-0.444)	
GI symptoms		Total: 17.6 (95%CI: 12.3-24.5)	15/59 (25,4)	6.8 (95%CI: 0.044-0.092)	
		60 studies and 4,243 patients			
		China: 16.1 (95%CI: 10.9-23.0)			
		53 studies and 4,198 patients			
		Other countries: 33.4 (95%CI: 15.2-58.3)			
		7 studies and 45 patients			
Anorexia		26.8 (95%CI: 16.2-40.8)			
18 studies
Nausea/vomiting	Total: 7.8 (7.1-8.5)	10.2 (95%CI: 6.6-15.3)	1/59 (1,7)		3.90
	26 studies and 5,955 patients	32 studies			
	China: 5.2 (4.4-5.9)				
	19 studies and 4,054 patients				
	Other countries: 14.9 (13.3-16.6)				
	7 studies and 1,901 patients				
Diarrhea	Total: 7.7 (7.2-8.2)	12.5 (95%CI: 9.6-16.0)	13/59 (22.0)	6.8 (95%CI: 0.044-0.092)	4.80
	43 studies and 10,676 patients				
	China: 5.8 (5.3-6.4)				
	32 studies and 8,612 patients				
	Other countries: 18.3 (16.6-20.1)				
	11 studies and 2,064 patients				
Abdominal pain	Total: 3.6 (3.0-4.3)	9.2 (95%CI: 5.7-14.5)	7/59 (11.9)		
	15 studies and 4,031 patients	12 studies			
	China: 2.7 (2.0-3.4)				
	10 studies and 2,447 patients				
	Other countries: 5.3 (4.2-6.6)				
	5 studies and 1,584 patients				
PCR in feces	15/50 (30)	48.1% (95%CI: 38.33-57.94)	9/59 (15.3)		
	7/10 (70)	12 studies and 138 patients			
	44/153 (29)				
Fever			56/59 (94.9)	87.3 (95%CI: 0.838-0.909)	88.50
Dry cough			22/59 (37.3)	58.1 (95%CI: 0.502-0.660)	68.60
Sore throat				12.0 (95%CI: 0.062-0.177)	
Productive cough				29.4 (95%CI: 0.171-0.417)	28.20
Chest pain				31.2 (95%CI: −0.024-0.648)	
Myalgia or fatigue				35.5 (95%CI: 0.253-0.456)	35.80
Headache or dizziness				9.4 (95%CI: 0.063-0.126)	12.20
Dyspnea			4/59 (6.8)	38.2 (95%CI: 0.246-0.520)	21.90

IQR: interquartile range; CI: confidence interval; M: men; GI: gastrointestinal.

Jin et al., did a retrospective study in China that analyzed 651 patients (331 men and 320 women), mean age of 46.14±14.19 years, with confirmed with COVID-19, in the Zhejiang province. Of these, 74 (11.4%) presented with at least one gastrointestinal symptom, such as nausea, vomiting, and diarrhea at hospital admission. The most common gastrointestinal symptom was diarrhea (8.14%), with a mean duration of 4 days, and most of the cases were self-limited. Of the patients with gastrointestinal symptoms, 29 had significantly higher rates of fever >38.5°C (39.19% *versus* 17.50% of those without gastrointestinal symptoms; p<0.001), 23 fatigue (31.08% *versus* 16.64%; p=0.004), eight dyspnea (10.81% *versus* 3.30%; p=0.007), and 16 headache (21.62% *versus* 8.84%; p=0.002). There was also a significant difference between patients with chronic liver disease (10.81% with gastrointestinal symptoms *versus* 2.95% without gastrointestinal symptoms; p=0.004) and severity of the disease at admission, with respiratory failure, and shock. In addition, multiple organ failure requiring mechanical ventilation and admission to the intensive care unit (ICU; 22.97% *versus* 8.14%; p<0.001). Regarding test findings, serum sodium levels were lower in patients with gastrointestinal symptoms (137.65mmol/L *versus* 138.33mmol/L; p=0.016), with a tendency towards having more severe disease. Aspartate aminotransferase (AST) had a higher rate in these patients (29.35U/L *versus* 24.4U/L; p=0.02). Patients with gastrointestinal symptoms had more significant complications during treatment, such as progression to SARS with need of ICU admission (6.67% *versus* 2.08%; p=0.034), and liver injury (17.57% *versus* 8.84%; p=0.035), than did those without gastrointestinal symptoms. The presence of viral RNA in feces was detected in few patients - only three of nine patients with gastrointestinal symptoms. Because of the small sample, they did not evaluate the implications of oral-fecal transmission, which needs further investigation. It is worth remembering, however, that this study had some limitations. It was not a cohort and did not identify patients with gastrointestinal symptoms who did not have the typical symptoms of cough and fever.^(^^18^^)^

In the study of Lin et al., 95 patients (50 women and 45 men) from Zhuhai, China, with COVID-19 and mean age of 45.3±18.3 years, 58 (61.0%) presented with gastrointestinal symptoms, in which 11 (11.6%) at hospital admission and 47 (49.5%) during hospitalization. The latter probably had the condition aggravated by use of drugs, such as antibiotics. The main gastrointestinal symptoms were diarrhea, anorexia, and nausea, in 24.2%, 17.9%, and 17.9%, respectively. The presence of RNA of the virus in feces was tested in 65 patients (42 with and 23 without gastrointestinal symptoms) and was positive in 22 (52.4%) of those with gastrointestinal symptoms, and 9 (39.1%) of those without. The presence of RNA of the virus in feces does not necessarily indicate more severe gastrointestinal symptoms, because it did not show a significant difference between the two groups (p=0.31). However, the presence of the virus in the tissue indicated a more severe disease. In all, six patients were submitted to endoscopy (two were severe and four non-severe), and one presented with bleeding in the esophagus with ulcers and erosions. The two severe patients were also submitted to proctoscopy, and the RNA of the virus was detected in the esophagus, stomach, duodenum, and rectum of these patients. Of the non-severe patients, only one had viral RNA presente in the duodenum. This study concluded that GIT can be a potential transmission route and target organ for SARS-CoV-2, and gastrointestinal symptoms cannot be underestimated.^(^^19^^)^

According to the descriptive, cross-sectional, multicenter study (three hospitals in Hubei, China) by Pan et al., with 204 patients, in which 107 were male, mean age of 52.91±15.98 years, 103 (50.5%) reported some gastrointestinal symptom, such as lack of appetite (81; 78.6%), diarrhea (35; 34.0%), vomiting (4; 3.9%), and abdominal pain (2; 1.9%). If we eliminate the lack of appetite, there were 38 (18.6%) patients with gastrointestinal symptoms. Six patients (3%) reported only digestive symptoms. It was noted that with increased severity of the disease, the gastrointestinal symptoms became more pronounced. Patients with gastrointestinal symptoms had more laboratory abnormalities, such as elevation of alanine aminotransferase (ALT; 42.24U/L *versus* 29.53U/L; p=0.011) and AST (35.12U/L *versus* 27.48U/L; p=0.032), decrease in monocyte count (0.39x10^9^/L *versus* 0.46x10^9^/L); p=0.021), increased prothrombin time (13.13 seconds *versus* 12.53 seconds; p=0.024), and received more antimicrobials during treatment (76.70% *versus* 61.39%; p=0.018), when compared to those without gastrointestinal symptoms. Thus, these patients had a greater chance of suffering hepatic injury. Yet, since this was a retrospective study, some limitations should be considered, such as small sample and they did not test the presence of SARS-CoV-2 in the patients’ feces.^(^^20^^)^

Wan et al., conducted a multicenter retrospective study with 14 hospitals in China and showed gastrointestinal symptoms are common in patients with COVID-19. Of 230 patients with the disease, 129 were men, mean age of 47.5 years, and diarrhea was observed in 49 (21%) of them. Other gastrointestinal symptoms, such as abdominal pain (3.1%) and melena (10.4%), were also reported. The patients with diarrhea had more comorbidities (39% *versus* 22%; p=0.017), more advanced age (55 years *versus* 46 years; p=0.017), and presented with more severe symptoms of respiratory problems, requiring ICU (31% *versus* 11%; p=0.0015), mechanical ventilation (12% *versus* 2%; p=0.0036), and progressing to death (8% *versus* 1%; p=0.020). The study has no data on asymptomatic patients, which may overestimate the prevalence of gastrointestinal symptoms in COVID-19.^(^^21^^)^

A cohort study by Redd et al., in nine hospitals in Massachusetts, United States, evaluated the presence of gastrointestinal symptoms in 318 adult patients (174 men) with COVID-19 at hospital admission and mean age 63.4±16.6 years. A total of 195 (61.3%) patients presented with gastrointestinal symptoms upon admission, among which were anorexia (110; 34.8%), diarrhea (107; 33.7%), nausea (84; 26.4%), and vomiting (49; 15.4%). The general symptoms significantly associated with the gastrointestinal tract were fatigue (65.1% *versus* 45.5%; p=0.0006), myalgia (49.2% *versus* 22.0%; p<0.0001), sore throat (21.5% *versus* 9.8%; p=0.0064), anosmia (13.3% *versus* 4.9%; p=0.0146), and ageusia (10.9% *versus* 2.4%; p=0.0057). A subgroup of 202 patients required hospitalization at the time this article was being written; 35 (17.5%) went to the ICU, 26 (13.0%) needed mechanical ventilation, and 32 (15.8%) died, but no significant difference was observed with worsening of the clinical picture in patients with and without gastrointestinal symptoms. The limitations of the study were a retrospective design, lack of instruments to validate symptoms, focus only on hospital data, and the fact that outpatients with less severe disease were excluded. In addition, the article did not evaluate the presence of the virus in the stool of these patients.^(^^22^^)^

Nobel et al., performed a retrospective control case study that compared the symptoms of 278 patients positive for SARS-CoV-2 with 238 negative patients in the United States. The gastrointestinal symptoms considered were diarrhea or nausea/vomiting. They found a significant difference between gastrointestinal symptoms in patients with and without COVID-19 (p=0.04). The presence of these symptoms was associated to a risk greater than 70% of having COVID-19. Among the COVID-19 patients, 97 (35%) presented with gastrointestinal symptoms, such as diarrhea (56; 20%) and nausea/vomiting (63; 23%). Patients with gastrointestinal symptoms had a longer duration of the disease when compared to those without these symptoms (33% *versus* 22%; p=0.048). The limitations of the study were a short patient follow-up period, a retrospective study, and the identification of gastrointestinal symptoms was dependent on documentation done well. This article also did not evaluate the presence of the virus in the feces of the patients.^(^^23^^)^

In a cohort study by Cholankeril et al., in an organization in the United States, gastrointestinal symptoms were reported in 31.9% (97) of patients with COVID-19, and the most common symptoms were loss of appetite (22; 25.3%), nausea/vomiting (12; 10.3%), and diarrhea (12; 10.3%). The study included 116 patients with COVID-19, most of them male (53.4%), mean age of 50 years. No patient presented with isolated gastrointestinal symptoms as the initial manifestation. The mean duration of gastrointestinal symptoms was one day, which was significantly shorter than the duration of respiratory symptoms (p<0.001). The elevation of AST correlated with severity of the disease (p=0.009). This study presented limitations, such as patients from only one organization (regional trend), the fact of documentation of extrapulmonary symptoms may be incomplete, and the diagnosis of COVID-19 was only done in patients with respiratory symptoms. The article did not evaluate the presence of the virus in the feces of these patients.^(^^24^^)^

For Zhang et al., who studied in 140 patients from China with COVID-19, 39.6% of them presented with gastrointestinal symptoms. In this study, the proportion between men and women was practically the same, and mean age was 57.0 years. The main symptoms were fever (91.7%), cough (75.0%), and fatigue (75.0%). They point out that eosinopenia, with or without lymphopenia in patients with symptoms and radiological changes, may be a potential indicator for the diagnosis of COVID-19. In addition, they found that allergic diseases, asthma, and chronic obstructive pulmonary disease (COPD) are not risk factors for SARS-CoV-2 infection, but studies with larger samples are needed to confirm these findings. Advanced age (p<0.001), comorbidities (p=0.002), and laboratory abnormalities are associated with severity of the disease. These laboratory values are high D-dimer (p<0.001), C-reactive protein (p<0.001), procalcitonin (p<0.001), and leukopenia (p<0.014). In this study, patients with COPD and smokers had a lower risk of being infected by SARS-CoV-2, but the progression of the condition in these patients was more severe. However, the relation of smokers with COVID-19 is still uncertain. Smokers and COPD patients are more susceptible to MERS-CoV infections, because tobacco and COPD increase the expression of the DPP4 carrier, which is the receptor for this virus.^(^^25^^)^

Zhou et al., evaluated 254 patients of a Wuhan center with confirmation of COVID-19. They compared the infected medical team separately from the non-medical patients. In the sample, 139 were women, mean age was 50.6 years, and 161 did not belong to the medical team. The most frequently reported symptoms in all patients were fever (211; 83%), cough (98; 38.6%), and gastrointestinal symptoms (66; 26%). In non-medical patients, the proportion of gastrointestinal symptoms in women was significantly higher than in men (62.8% *versus* 37.2%; p=0.033). In addition, clinical manifestations, such as sore throat (14% *versus* 3%; p=0.023), dizziness (12% *versus* 3%; p=0.032), fatigue (67% *versus* 44%; p=0.012), low hemoglobin (116.7 *versus* 133; p=0.028), high C-reactive protein (7.3 *versus* 3.8; p=0.021), and high ALT (64.1 *versus* 46.6; p=0.049), were also significantly more frequent in non-medical patients with gastrointestinal symptoms. Nonetheless, there was no significant correlation between symptoms and clinical characteristics of patients with and without gastrointestinal symptoms. An explanation of why the medical team was less susceptible to gastrointestinal symptoms is that most of this group was made up of young nurses with no comorbidities. This study has limitations. Most of the cases included were clinically confirmed, therefore it has patients without the PCR test for SARS-CoV-2. Additionally, many patients did not have follow-up, since they were still hospitalized at the time of submission of this article.^(^^26^^)^

Cipriano et al., conducted a systematic review with six studies of patients from China, which points to the possibility of SARS-CoV-2 infection in the gastrointestinal tract and fecal-oral transmission. In the study, 53.42% of stool samples tested positive for the presence of SARS-CoV-2 RNA in 73 hospitalized patients. These samples remained positive between one and 12 days, and 23.29% of patients had feces positive for the virus RNA after the respiratory samples tested negative. The authors conclude that the anal swab can be as important as the nasopharyngeal swab, even in asymptomatic patients. Before hospital discharge, physicians should consider that gastrointestinal infection and potential fecal-oral transmission may remain until after the viral disappearance of the respiratory tract.^(^^30^^)^

Cheung et al., did an interesting study on gastrointestinal symptoms of COVID-19 and the presence of viral RNA in feces. They compared a meta-analysis with a Hong Kong cohort. In a cohort of 59 patients with the disease, 15 (25.4%) presented with gastrointestinal symptoms, and 9 (15.3%) had their feces tested positive for viral RNA; 38.5% and 8.7% of those with and without diarrhea, respectively (p=0.019), had stool viral RNA detected. In the meta-analysis, which included 60 studies and 4,243 patients, the prevalence of patients with gastrointestinal symptoms was 17.6%; in that, 16.1% in studies from China, and 33.4% from other countries (South Korea, Singapore, Vietnam, United States, and United Kingdom). The gastrointestinal symptoms evaluated were anorexia (26.8%), diarrhea (12.5%), nausea/vomiting (10.2%), and abdominal pain (9.2%). The prevalence of RNA of the virus in feces was 48.1%. Of these samples, 70.3% tested positive in the feces and could persist for more than 33 days from the onset of symptoms, even after the respiratory system had resulted negative for viral RNA. This meta-analysis also showed the prevalence of severe disease was more common in patients with gastrointestinal symptoms than in those without (17.1% *versus* 11.8%). The study limitations are underreporting of gastrointestinal symptoms and fewer studies outside China. Thus, healthcare professionals should pay attention to stool collection and procedures, such as endoscopy in patients with COVID-19.^(^^27^^)^

The systematic review and meta-analysis with 31 articles and 46,959 patients from China, by Cao et al., described the prevalence of symptoms such as fever (87.3%), dry cough (58.1%), dyspnea (38.2%), myalgia or fatigue (35.5%), chest pain (31.2%), productive cough (29.4%), sore throat (12%), headache (9.4%), and diarrhea (6.8%). The incidence of ICU use, SARS, multiple organ failure, and mortality was 29.3%, 28.8%, 8.5%, and 6.8%, respectively. The limitations of these studies were no foreigners included and no comments on viral presence in the feces.^(^^28^^)^

The meta-analysis made by Li et al., with 10 articles and 1,994 patients from China, showed similar results. The main symptoms reported were fever (88.5%), cough (68.6%), myalgia or fatigue (35.8%), productive cough (28.2%), and dyspnea (21.9%). Other symptoms were headache or dizziness (12.1%), diarrhea (4.8%), and nausea and vomiting (3.9%). Lymphopenia (64.5%), increased C-reactive protein (44.3%), increased lactate dehydrogenase (28.3%), and leukopenia (29.4%) were also recorded. In addition, men were more affected (60%), and the fatality rate was 5%. This study presented limitations by the number and quality of studies included.^(^^29^^)^

Sultan et al., performed a systematic review and meta-analysis of 47 studies with 10,890 patients. The prevalence of diarrhea was 7.7%, nausea and vomiting 7.8%, and abdominal pain 2.7%. When comparing data from China and other countries, diarrhea (5.8% *versus* 18.3%), nausea and vomiting (5.2% *versus* 14.9%), abdominal pain (2.7% *versus* 5.3%), and liver abnormalities were more prevalent outside China. The liver alterations studied were AST elevation (total: 15.0%; China: 14.9%; other countries: 20.0%), ALT (total: 15.0%; China: 14.9%; other countries: 19.0%), and bilirubin (total or China: 16.7%). There was no deeper analysis about the presence of viral RNA in the feces of these patients, but the authors found a variable prevalence of 15/50 (30%), 7/10 (70%), and 44/153 (29%). The authors also provided a manual of recommendations for good practices in the management of COVID-19 with seven items; the main points are to obtain a detailed history of symptoms (typical and GIT), to verify liver function values upon admission of patients and monitor during hospitalization, and to evaluate the adverse reactions of drugs used in treatment. Regarding limitations, the patients were hospitalized (prevalence may be under or overestimated), the symptoms may have been analyzed inconsistently, information from laboratory tests was lacking, and there was no information about gastrointestinal symptoms being systematically evaluated at admission, among others. They concluded hepatic enzyme monitoring may be beneficial, and there is still no evidence to support the viral PCR test in the stools as a diagnosis or for monitoring of COVID-19 as a routine in clinical practice, because the results of other meta-analyses are conflicting and further studies on the subject are needed.^(^^8^^)^

## DISCUSSION

With the evaluation of all these studies, one can see that there is still a lot of variation in the results regarding several points, and the factors studied were very different.

The mean age of patients ranged from 45.1 to 58.5 years, but only one article significantly correlated advanced age with gastrointestinal symptoms.^(^^21^^)^ In another article, advanced age was related to severity of the disease, but without assessing whether these patients had gastrointestinal symptoms.^(^^25^^)^ In general, all articles found more men affected with COVID-19 than women, and only one found a significant difference between non-medical women and more gastrointestinal symptoms.^(^^26^^)^ These results suggested that advanced age and male gender are risk factors for infection. The ACE2 receptor gene, target of SARV-CoV-2, is located on the X chromosome. A study still under review found that ACE2 expression increases in females and with sex hormones (which decrease with ageing); additionally, inflammatory cytokines decrease this expression (which increase with ageing and chronic diseases). This is contrary to what is currently thought, that is, increased ACE2 is to be blamed for the worse prognosis of COVID-19. SARS-CoV-2 binds directly to ACE2, causing downregulation of this receptor, which is even less in males, for example, and may explain the worse prognosis and the more prevalent infection in them.^(^^31^^)^

Regarding the comorbidities of infected patients, the most often cited were hypertension, *diabetes mellitus*, cerebrovascular disease, cardiovascular disease, lung disease, kidney disease, liver disease, and cancer, among others. However, only three articles reported a significant relation between gastrointestinal symptoms and comorbidities, and one of them ins chronic liver disease.^(^^18^^,^^21^^,^^25^^)^ On the other hand, the relation between COVID-19 and comorbidities also proved to be diverse. Very frequent comorbidities in the population were addressed and may have important significance in SARS-CoV-2 infection and prognosis.

The typical symptoms most often described are common in respiratory tract infections, such as fever (76.7% to 94.9%), dry cough (38.6% to 77.7%), productive cough (14.2% to 43.0%), fatigue (16.6% to 75.0%), dyspnea (3.3% to 60.1%), headache (8.0% to 21.6%), and sore throat (8.11% to 17.0%).^(^^8^^,^^18^^-^^29^^)^ Other symptoms, such as anosmia and ageusia, are closely related to COVID-19, but only one study identified their presence (10.1% and 7.6%, respectively). This study found a correlation between the presence of fatigue, myalgia, dyspnea, sore throat, anosmia, and ageusia with gastrointestinal symptoms.^(^^22^^)^ Two other studies also identified relation between gastrointestinal symptoms and typical symptoms; one with fever above 38.5°C, fatigue, dyspnea, and headache, and the other with fatigue, sore throat, and dizziness.^(^^18^^,^^26^^)^

Regarding gastrointestinal symptoms, the prevalence showed great variation, ranging between 6.8% and 61.3% in these studies. This shows that there may be a regional trend in them, and studies with many patients from several countries are warranted to improve this relation between GIT and COVID-19. It is important to remember that two meta-analyses analysed China and other countries, and showed that the other sites presented with higher rates of gastrointestinal symptoms.^(^^8^^,^^27^^)^ However, there were few other countries, and many were in Asia. Nevertheless, this shows that gastrointestinal symptoms are important in this disease, may have a great impact in some countries, and should be evaluated based on history of suspected patients. The most often cited gastrointestinal symptoms were diarrhea (8.14% to 33.7%), nausea/vomiting (1.53% to 26.4%), anorexia (12.1% to 40.0%), and abdominal pain (0% to 14.5%).^(^^8^^,^^18^^-^^29^^)^

Several studies point out relations between laboratory changes and gastrointestinal symptoms or severity of the disease. Liver injury (elevation of AST, ALT, and bilirubin) was the most often evaluated, but elevations of C-reactive protein, procalcitonin, lactate dehydrogenase, D-dimer, and prothrombin were also verified, as well as a decrease in serum sodium levels, leukocytes, lymphocytes, and monocytes.^(^^8^^,^^18^^,^^20^^,^^24^^,^^25^^,^^29^^)^ Hence, it is very important to evaluate these parameters in patients hospitalized with COVID-19, since they may indicate a worse prognosis of the disease.

An important factor infrequently evaluated among the studies was the presence of viral RNA in stool samples from patients infected with SARS-CoV-2. This data would help us to understand if there is a possibility of fecal-oral transmission in this disease. Only four studies performed this analysis, in which two were meta-analyses and two retrospective studies. This test was positive in 0% to 48.1% of cases, with great heterogeneity in the results, but patients showing gastrointestinal symptoms presented more frequently the presence of virus RNA in feces.^(^^8^^,^^18^^,^^19^^,^^27^^)^ Some also identified that this result remained positive even after the negativity of respiratory tract samples. In one of them, 53.42% of feces samples were positive for the virus, and 23.29% were still positive when the respiratory tract was negative.^(^^30^^)^ In another, 70.3% of patients who had feces positive for the virus (48.1%) also remained positive after respiratory clearance.^(^^27^^)^ However, studies did not evaluate whether the RNA present in feces represents infecting virus or only viral residues, with no capacity to infect. These data lead us to doubt whether the PCR test in feces of patients with COVID-19 should be done routinely or not. Therefore, further studies should be concerned and evaluate this issue, as possible fecal-oral transmission can have a major impact in developing countries that lack basic sanitation in many places.

Finally, the need for ICU admission ranged from 2.08% to 31.0%, and cases that had evolved to death from 0% to 17.65%. Several studies have commented on this, but only two presented with a significant difference between the presence of gastrointestinal symptoms and the need for ICU, or death.^(^^8^^,^^21^^)^ Thus, the investigation of these patients with gastrointestinal symptoms since the onset of the infectious condition becomes very important, considering many of them, more than those without these symptoms, may develop a more serious disease and need ICU, or even progress to death.

The articles in this review have several limitations. In general, the evaluation of the symptoms was done differently among them, there may be underreporting of gastrointestinal symptoms, and few were carried out with patients outside China. Additionally, the design was retrospective and had small samples, many did not evaluate the presence of viral RNA in feces, did not include asymptomatic patients, the data were hospital data, and the follow-up of patients was short. Moreover, because this review includes different study designs, there is a risk that the meta-analyses and systematic reviews also contain some retrospective article and may have duplicate results. The various study designs, *per se*, are also a limitation of this review, since the ideal would be to use the same model.

## COMMENTS

The high rates of SARS-CoV-2 infection worldwide have caused the World Health Organization to declare a pandemic in 2020. Therefore, information about this new disease is of extreme importance, both in terms of pathophysiology and prevalence, severity, risk factors, diagnosis, and treatment. With this effort, we realize that the gastrointestinal tract can be greatly influenced by the disease, causing specific symptoms (diarrhea, abdominal pain, nausea, and vomiting) and laboratory changes (mainly of hepatic enzymes). Health professionals should pay attention to this, keeping alert to the modifications that can help in the diagnosis, and initiate early treatment in order to avoid bad prognosis. However, the results of prevalence of the gastrointestinal symptoms were very different, presenting a variation from 6.8% to 61.3%. These symptoms were diarrhea (8.14% to 33.7%), nausea/vomiting (1.53% to 26.4%), anorexia (12.1% to 40.0%), and abdominal pain (0% to 14.5%). This important variation demands larger studies, with more patients and from various countries. As to the possibility of fecal-oral transmission, the presence of viral RNA was tested little, but was positive between 0% and 48.1%. These data also varied a lot, preventing any conclusion about them, but they should also be studied further so that preventive measures can be taken. The RNA of the virus in feces does not necessarily indicate that there is fecal-oral transmission, and its evaluation in new studies to know if this presence is infectious or not is very important.
