# Supplementary material for: COVID-19 and the gastrointestinal tract: what do we already know?
Source: Einstein (Sao Paulo). 2020 Nov 5;18:eRW5909. doi: 10.31744/einstein_journal/2020RW5909 (PMC7647386; doi:10.31744/einstein_journal/2020RW5909)
Supplement: Translation

# Translation: COVID-19 e o trato gastrintestinal: o que já sabemos?

**Authors:** Joana Ferro Machado de Almeida, Ethel Zimberg Chehter

**Affiliations:** 1 Santo AndréSP Brasil Faculdade de Medicina do ABC, Santo André, SP, Brasil.

**Keywords:** SARS-CoV-2, Betacoronavírus, Infecções por coronavírus, COVID-19, Trato gastrointestinal, Doenças gastrointestinais/etiologia

## Abstract

Com quase 17,5 milhões de infectados e 700 mil mortos até o início de agosto no mundo, a pandemia do novo coronavírus está marcando o ano de 2020. O agente causador da doença é o vírus SARS-CoV-2, e a transmissão é por via respiratória. Os infectados podem ser assintomáticos, apresentar sintomas típicos (febre, tosse seca e dispneia), sintomas gastrintestinais (diarreia, náusea, vômito e dor abdominal) e RNA viral nas fezes. O objetivo deste trabalho foi revisar a literatura relacionada com a prevalência dos sintomas gastrintestinais, e verificar se é possível a transmissão fecal-oral da doença. Fizemos uma pesquisa na base de dados PubMed^®^ sobre a COVID-19 e o trato gastrintestinal, selecionando artigos pelo método PRISMA. Eliminamos artigos com base em títulos e resumos, quantidade pequena de pacientes e sobre mecanismo de infecção, restando 14 estudos. Comorbidades e alterações laboratoriais (elevação de aminotransferases hepáticas e bilirrubina) foram relacionadas com piora da doença. A prevalência de sintomas gastrintestinais variou entre 6,8% e 61,3%, sendo eles diarreia (8,14% a 33,7%), náusea/vômito (1,53% a 26,4%), anorexia (12,1% a 40,0%) e dor abdominal (0% a 14,5%). A presença do RNA viral foi pouco testada, mas foi positiva entre 0% a 48,1%. O trato gastrintestinal é muito acometido pela COVID-19, provocando sintomas específicos, alterações laboratoriais e presença viral nas fezes. Contudo, os resultados de prevalência e a possibilidade de transmissão fecal-oral foram variados, necessitando de estudos maiores para conclusões mais assertivas. É importante a atenção de profissionais da saúde a isso, visto que essas alterações podem ajudar no diagnóstico e a iniciar tratamento precoce.

## INTRODUÇÃO

Em dezembro de 2019, foram identificados os primeiros casos de pacientes infectados com o novo coronavírus em Wuhan, na China.^(^^1^^)^ O ano de 2020 está sendo marcado pela pandemia da doença pelo coronavírus 2019 (COVID-19), causando grande impacto na saúde pública e na economia de diversos países. Em 1º de agosto, segundo o site da Organização Mundial da Saúde (OMS), havia 17.396.943 casos acumulados e 675.060 mortes no mundo. O país com maior número de infectados e mortes é os Estados Unidos, com 4.456.389 e 151.265, respectivamente. No Brasil, esses números eram de 2.610.102 casos e 91.263 mortes.^(^^2^^)^

O vírus, chamado de coronavírus da síndrome respiratória aguda grave 2 (SARS-CoV-2), é o sétimo coronavírus que, sabidamente, infecta humanos. Os demais são o coronavírus da síndrome respiratória aguda grave (SARS-CoV), cuja epidemia ocorreu em 2003, e o coronavírus da síndrome respiratória do Oriente Médio (MERS-CoV), cuja epidemia ocorreu no Oriente Médio em 2012, os quais causam doença severa, e HKU1, NL63, OC43 e 229E, que provocam sintomas leves. O SARS-CoV-2 é um vírus RNA encapsulado da ordem *Nidovirales*, família *Coronaviridae*, subfamília *Coronavirinae*, gênero Beta.^(^^3^^)^ Provavelmente, o SARS-CoV-2 foi originado de morcegos e transmitido para humanos por meio de outro animal.^(^^4^^)^ A principal via de transmissão é respiratória, por meio de contato com gotículas, aerossóis e superfícies contaminadas.^(^^5^^)^

Os sintomas típicos da doença são febre, tosse seca, dispneia, cefaleia, anosmia, disgeusia e pneumonia, mas ela também pode ser assintomática. Pode provocar insuficiência respiratória progressiva, por meio de lesão alveolar, podendo levar à morte.^(^^1^^,^^6^^)^ Entretanto, diversos estudos apontam que os pacientes podem apresentar sintomas relacionados com o trato gastrintestinal (TGI) e a presença do vírus em células epiteliais do TGI e de seu RNA nas fezes. Com base nisso, uma possível transmissão oral-fecal da doença é apontada.^(^^6^^,^^7^^)^ Os principais sintomas gastrintestinais são diarreia, náusea, vômitos e dor abdominal.^(^^8^^)^

O vírus SARS-CoV-2 utiliza a enzima conversora de angiotensina 2 (ECA2) como receptor para sua entrada nas células. Ele infecta principalmente as células pulmonares, mas pode ter acometimento gastrintestinal, que também possui receptores ECA2 na borda em escova da mucosa intestinal.^(^^6^^)^ Além disso, o vírus é liberado na parte apical das células pulmonares. Com isso, ele é arrastado pelo movimento mucociliar e pode ganhar o TGI. Esses receptores ECA2 agem diminuindo a ação do sistema renina-angiotensina-aldosterona, ao metabolizar a angiotensina 2. Em níveis séricos altos, a angiotensina 2 tem efeitos nos sistemas cardiovascular, renal e respiratório, podendo causar hipertensão crônica e falência renal e pulmonar. O vírus SARS-CoV-2 provoca *downregulation* (regulação negativa) e bloqueio desses receptores ECA2, explicando o quadro clínico de falência pulmonar e síndrome de dificuldade respiratória do adulto em alguns pacientes infectados.^(^^9^^)^

A superfície viral possui glicoproteínas S (*Spike*), que precisam ser separadas em domínios S1 e S2, para que o vírus consiga aderir à membrana celular. Furina e serina protease transmembrana (TMPRSS2) são substâncias que possibilitam essa separação. Assim, a S1 se liga ao receptor ECA2 e a S2 à membrana celular, possibilitando a entrada do vírus na célula por meio de endocitose. Furina e TMPRSS2 são enzimas presentes nas células do intestino delgado, sendo que a primeira também tem ação na ativação de toxinas de alguns microrganismos.^(^^7^^,^^9^^)^

A ECA2 da mucosa intestinal está associada com o transportador de aminoácidos B^0^AT1 e regula a flora intestinal. Isso ocorre porque esse transportador permite a absorção de triptofano, que estimula a via mTOR a produzir peptídeos antimicrobianos. Assim, a infecção pelo SARS-CoV-2 altera a quantidade e bloqueia os receptores ECA2 na borda em escova, causando deficiência de triptofano e menor produção de peptídeos antimicrobianos, podendo causar alteração do microbioma intestinal e inflamação.^(^^7^^,^^9^^)^

Pacientes com COVID-19 apresentam grande reação inflamatória, causada pela chamada tempestade de citocinas, que pode ser originada ou potencializada pelo TGI. O intestino delgado possui a maior quantidade de tecido linfoide do organismo, com placas de Peyer, linfonodos mesentéricos e folículos linfoides ao longo do intestino. Os intestinos também possuem, na mucosa e abaixo da lâmina própria, grande população de células T ativadas, células plasmáticas, mastócitos, células dendríticas e macrófagos. No cenário de uma infecção como a COVID-19, há liberação exagerada de citocinas, que promovem o recrutamento de diversas células, causando grande processo inflamatório.^(^^9^^,^^10^^)^ Estudos apontam que o interferon e a infecção pelo vírus influenza (indutor da via de interferon) podem estar relacionados com aumento de transcrição de ECA2.^(^^11^^)^ Fumantes e pacientes com doença pulmonar obstrutiva crônica (DPOC) também têm maior expressão de receptores ECA2.^(^^12^^)^ Além disso, diabetes e hipertensão têm sido associados com polimorfismos do gene da ECA2.^(^^7^^)^

Medicamentos utilizados no tratamento de hipertensão, como inibidores da ECA (IECA) e bloqueadores dos receptores de angiotensina (BRA), reduzem inflamação pela diminuição de citocinas. Além disso, eles podem aumentar a expressão da ECA2.^(^^13^^)^ Entretanto, o uso de IECA e BRA não foi associado com pior evolução clínica em pacientes com COVID-19. Assim, recomenda-se a manutenção do uso desses medicamentos em pacientes hipertensos.^(^^14^^,^^15^^)^

Enquanto os vírus SARS-CoV e SARS-CoV-2 utilizam a ECA2 como mediadora para entrar nas células, o MERS-CoV utiliza a dipeptidil-peptidase 4 (DPP4).^(^^7^^)^ Por serem vírus da mesma família, alguns autores passaram a estudar se a DPP4 também está envolvida no mecanismo de infecção da nova doença. De acordo com Vankadari et al., o domínio S1 da glicoproteína de membrana do SARS-CoV-2, além de interagir com a ECA2, que é a principal porta de entrada para o novo vírus, pode ter relação com a DPP4.^(^^16^^)^ Entretanto, estudo de Tai et al., verificou que o vírus não se ligou a células 293T que expressavam apenas DPP4. Essa ligação ocorreu apenas em células 293T com ECA2.^(^^17^^)^

Diversos artigos sobre os sintomas da COVID-19 relacionados ao TGI e sua possível transmissão oral-fecal têm sido publicados, com resultados muito divergentes.

O objetivo deste estudo foi revisar a literatura relacionada com a prevalência dos sintomas gastrintestinais e verificar se há a possibilidade de transmissão fecal-oral da doença.

## MÉTODOS

A base de dados escolhida para esse trabalho foi o PubMed^®^. No dia 5 de maio de 2020, procuramos artigos com as palavras “COVID-19” ou “SARS-CoV-2” e “TGI” ou “gastrointestinal” ou “entérico”. Dessa pesquisa, resultaram 179 artigos. Os critérios de inclusão desses estudos nesta revisão foram preenchimento dos critérios de elegibilidade (artigos que abordassem a infecção pelo SARS-CoV-2 e sintomas do TGI), artigos em inglês e amostragem com mais de 50 pacientes. Já os critérios de exclusão foram desenho de estudo de relatos de casos, revisões sobre o mecanismo de infecção e o vírus, manuais de recomendações de procedimentos relacionados com o TGI em pacientes infectados, artigos em idiomas diferentes do inglês e com menos de 50 pacientes avaliados. A figura 1 mostra o diagrama *Preferred Reporting Items for Systematic Reviews and Meta-Analyses* (PRISMA) realizado com base em leitura de títulos e resumos; 161 artigos foram eliminados, restando 18 artigos. Um último artigo foi adicionado posteriormente. Desses 19 artigos, quatro eram revisões sobre o vírus e seu mecanismo de infecção, sendo desconsiderados. Por fim, foi eliminado um estudo que incluíra apenas dez pacientes. Com isso, essa revisão foi feita com 14 artigos, sendo quatro metanálises, uma revisão sistemática e nove retrospectivos. Essa análise dos artigos foi feita inicialmente pelo autor, mas a eleição final contou também com um revisor sênior. Devido à pouca quantidade de estudos grandes relacionados a esse assunto e à importância e à urgência do tema, foram incluídas diversas variações de desenhos de estudo. As informações sobre país de origem, média de idade, diferentes comorbidades, sintomas típicos (febre, tosse, dispneia, entre outros), sintomas gastrintestinais (diarreia, náusea, vômito e dor abdominal) e presença de RNA viral nas fezes, quando citadas, foram incluídas neste estudo para análise.

**Figura 1:** Diagrama *Preferred Reporting Items for Systematic Reviews and Meta-Analyses* (PRISMA)

## RESULTADOS

Em relação aos estudos retrospectivos, três foram feitos apenas com pacientes dos Estados Unidos e seis da China. A revisão sistemática incluiu vários estudos conduzidos na China. Sobre as metanálises, duas incluíram estudos realizados apenas na China e duas avaliaram diversos países. As tabelas 1 a 3 apresentam os dados gerais, sintomas típicos e sintomas gastrintestinais de pacientes com COVID-19 dos estudos retrospectivos, respectivamente. A tabela 4 apresenta os sintomas dos pacientes com a doença nas metanálises.[Table t6]

**Tabela 1:** Dados gerais dos pacientes com COVID-19 dos nove estudos retrospectivos

	Jin et al.^(^^18^^)^			Lin et al.^(^^19^^)^				Pan et al.^(^^20^^)^			
	Com sintomas GI	Sem sintomas GI	Valor de p	Total	Com sintomas GI	Sem sintomas GI	Valor de p	Total	Com sintomas GI	Sem sintomas GI	Valor de p
Pacientes	74	577		95	58	37		204	103	101	
Idade	46,14±14,19	45,09±14,45	0,559	43,3±18,3	48,0±17,1	41,1±19,5	0,073	52,91±15,98	52,21±15,92	53,61±16,10	0,533
Sexo	H: 37/74 (50,0) M: 37/74 (50,0)	H: 294/577 (51) M: 283/577 (49)	H: 0,902	H: 45/95 (47,4) M: 50/95 (52,6)	H: 27/58 (46,6) M: 31/58 (53,4)	H: 18/37 (48,6) M: 19/37 (51,4)	M: 0,84	H: 107/204 (52,5) M: 97/204 (47,5)	H: 55/103 (53,4) M: 48/103 (46,6)	H: 52/101 (51,5) M: 49/101 (48,5)	0,784
IMC, kg/m^2^											
Tabaco	3/74 (4,23)	38/577 (6,59)	0,610	6/95 (6,3)	5/58 (8,6)	1/37 (2,7)	0,40				
Álcool				9/95 (9,5)	6/58 (10,3)	3/37 (8,1)	1,00				
Alguma comorbidade	25/74 (33,78)	153/577 (26,52)	0,212								
Doença coronariana											
Insuficiência cardíaca congestiva											
Arritmia											
Hipertensão	12/74 (16,22)	88/577 (15,25)	0,864	16/95 (16,8)	10/58 (17,2)	6/37 (16,2)	0,90				
*Diabetes mellitus*	7/74 (9,46)	41/577 (7,11)	0,477	6/95 (6,3)	3/58 (5,2)	3/27 (8,1)	0,67				
Doença cerebrovascular				4/95 (4,2)	3/58 (5,2)	1/37 (2,7)	1,00				
Doença pulmonar	0/74 (0)	1/577 (0,17)	1,00	5/95 (5,3)	1/58 (1,7)	4/37 (10,8)	0,074	9/204 (4,41)	7/103 (6,80)	2/101 (1,98)	0,182
Doença renal crônica	0/74 (0)	6/577 (1,04)	1,00	1/95 (1,1)	1/58 (1,7)	0/37 (0)	1,00				
Doença hepática crônica	8/74 (10,81)	17/577 (2,95)	0,004					1/204 (0,97)			
Câncer	0/74 (0)	6/577 (1,04)	1,00	5/95 (5,3)	4/58 (6,9)	1/37 (2,7)	0,65	13/204 (6,37)	8/103 (7,77)	5/101 (4,95)	0,410
Doença cardiovascular	1/74 (1,35)	4/577 (0,69)	0,454					44/204 (21,57)	23/103 (22,3)	21/101 (20,79)	0,789
Imunossupressão	0/74 (0)	1/577 (0,17)	1,00								
RNA viral nas fezes	3/9 (33,33)	0/577 (0)		31/65 (47,7)	22/42 (52,4)	9/23 (39,1)	0,31				
Suplementação de oxigênio											
UTI	5/74 (6,76)	12/577 (2,08)	0,034					16/204 (7,84)	6/103 (5,94)	10/101 (9,90)	0,279
Ventilação mecânica	5/74 (6,76)	12/577 (2,08)	0,034								
Morte				0/95 (0)	0/58 (0)	0/37 (0)		36/204 (17,65)	19/103 (18,45)	17/101 (16,83)	0,762

	Wan et al.^(^^21^^)^				Redd et al.^(^^22^^)^				Nobel et al.^(^^23^^)^	Cholankeril et al.^(^^24^^)^
	Total	Com sintomas GI	Sem sintomas GI	Valor de p	Total	Com sintomas GI	Sem sintomas GI	Valor de p	Total	Total
Pacientes	230	49	181		318	195	123		278	116
Idade	47,5 (36,8-60)	55 (40-65)	46 (36-57)	0,017	63,4±16,6	62,3±15,9	65,0±17,6	0,16	18-30: 31/278 (11)	50 (35-67)
									31-50: 69/278 (25)	
									51-70: 103/278 (37)	
									>70: 75/278 (27)	
Sexo	H: 129/230 (56) M: 101/230 (44)	H: 27/49 (55) M: 22/49 (45)	H: 102/181 (56) M: 79/181 (22)	0,87	H: 174/318 (54,7) M: 144/318 (45,3)	H: 102/195 (52,3) M: 93/195 (47,7)	H: 72/123 (58,6) M: 51/123 (35,4)	M: 0,28	H: 145/278 (52) M: 133/278 (48)	H: 62/116 (53,4) M: 54/116 (46,6)
IMC, kg/m^2^					30,0±6,5	30,5±6,7	29,3±6,2	0,11	<25,0: 25/278 (9)	25,8 (23,2-31,5)
									25,0-29,9: 43/278 (16)	
									≥30,0: 47/278 (17)	
									Sem avaliação: 165/278 (59)	
Tabaco					35/318 (11,0)	23/195 (11,8)	12/123 (9,8)	0,57		Ex-fumante: 21 (23,3)Fumante: 3 (2,8)
Álcool					39/318 (12,3)	28/195 (14,4)	11/123 (8,9)	0,15		
Alguma comorbidade	58/230 (25)	19/49 (39)	39/181 (22)	0,017						
Doença coronariana					46/318 (14,5)	26/195 (13,3)	20/123 (16,3)			
Insuficiência cardíaca congestiva					31/318 (9,8)	16/195 (8,3)	15/123 (12,2)	0,25		
Arritmia					48/318 (15,1)	26/195 (13,3)	22/123 (17,9)	0,27		
Hipertensão	40 (17)				188/318 (59,1)	111/195 (56,9)	77/123 (62,6)	0,32		32 (27,8)
*Diabetes mellitus*	11 (5)				105/318 (33,1)	62/195 (32,0)	43/123 (35,0)	0,58		19 (16,4)
Doença cerebrovascular					11/318 (3,5)	8/195 (4,1)	3/123 (2,4)	0,42		
Doença pulmonar (%)	7 (3)				67/318 (21,1)	40/195 (20,5)	27/123 (22,0)	0,76		24 (20,7)
Doença renal crônica					40/318 (13,8)	20/195 (11,7)	20/123 (17,0)	0,20		4 (3,5)
Doença hepática crônica	6 (3)									3 (2,8)
Câncer	3 (1)									
Doença cardiovascular	10 (4)									15 (12,9)
Imunossupressão										4 (4,3)
RNA viral nas fezes										
Suplementação de oxigênio		44/49 (90)	143/181 (79)	0,10						
UTI		15/49 (31)	20/181 (11)	0,0015	35/202 (17,5)	20 (15,4)	15 (21,4)	0,28	44/278 (16)	
Ventilação mecânica		6/49 (12)	3/181 (2)	0,0036	26/202 (13,0)	14 (10,9)	12 (16,0)	0,22		
Morte		4/49 (8)	2/181 (1)	0,020	32/202 (15,8)	16 (12,2)	16 (22,5)	0,06	9/278 (3,2)	
	**Zhang et al.**^(^^25^^)^	**Zhou et al.**^(^^26^^)^								
	**Total**	**Total**	**Total equipe médica**	**Com sintomas** **GI equipe médica**	**Sem sintomas** **GI equipe médica**	**Valor de p**	**Total não médicos**	**Com sintomas GI não médicos**	**Sem sintomas GI não médicos**	**Valor de p**
Pacientes	140	254	93	23	70		161	43	118	
Idade	57 (25-87)	50 (36-65)	36 (31-41)	35 (30-40)	36 (31-42)	0,614	62 (49-69)	61 (49-67)	62 (49-70)	0,615
Sexo	H: 71/140 (50,7) M: 69/140 (49,3)	H: 115/254 (45,3) M: 139/254 (54,7)	H: 32/93 (34) M: 61/93 (66)	H: 6/23 (26) M:17/23 (74)	H: 26/70 (37) M:44/70 (63)	0,45	H: 83/161 (52) M: 78/161 (48)	H: 16/43 (37) M: 27/43 (63)	H: 67/118 (57) M: 51/118 (43)	0,033
IMC, kg/m^2^										
Tabaco	Ex-fumante: 7/140 (5,0) Fumante: 2/140 (1,4)									
Álcool										
Alguma comorbidade	90/140 (64,3)									
Doença coronariana	7/140 (5,0)	17/254 (6,7)	2/93 (2,1)	0/23 (0)	2/70 (3)	1,00	15/161 (9)	6/43 (14)	9/118 (8)	0,231
Insuficiência cardíaca congestiva										
Arritmia	5/140 (3,6)									
Hipertensão	42/140 (30,0)	63/254 (24,8)	6/93 (6,4)	0/23 (0)	6/70 (9)	0,33	57/161 (35)	14/43 (33)	43/118 (36)	0,712
*Diabetes mellitus*	17/140 (12,1)	26/254 (10,2)	3/93 (3,2)	0/23 (0)	3/70 (4)	0,572	23/161 (14)	4/43 (9)	19/118 (16)	0,321
Doença cerebrovascular	3/140 (2,1)	13/254 (5,1)	1/93 (1,1)	0/23 (0)	1/70 (1)	1,00	12/161 (7)	3/43 (7)	9/118 (8)	1,00
Doença pulmonar	2/140 (1,4)	6/254 (2,4)	1/93 (1,1)	0/23 (0)	1/70 (1)	1,00	5/161 (3)	2/43 (5)	3/118 (3)	0,61
Doença renal crônica	2/140 (1,4)									
Doença hepática crônica		3/254 (1,2)	1/93 (1,1)	0/23 (0)	1/70 (1)	1,00	2/161 (1)	0/43 (0)	2/118 (2)	1,00
Câncer		2/254 (0,8)	1/93 (1,1)	0/23 (0)	1/70 (1)	1,00	1/161 (1)	0/43 (0)	1/118 (1)	1,00
Doença cardiovascular										
Imunossupressão		1/254 (0,4)	0/93 (0)	0/23 (0)	0/70 (0)		1/161 (1)	1/43 (2)	0/118 (0)	0,267
RNA viral nas fezes										
Suplementação de oxigênio										
UTI										
Ventilação mecânica		18/254 (7)	5/93 (5,4)	1/23 (4,3)	4/70 (5,7)	1,00	13/161 (8)	2/43 (4,7)	11/118 (9,3)	0,516
Morte		16/254 (6,3)	2/93 (2,1)	1/23 (4,3)	1/70 (1,43)	0,435	14/161 (9)	3/43 (7)	11/118 (9,3)	0,457

Resultados expressos como n, média±desvio padrão, n/n total (%).

GI: gastrintestinal; H: homens; M: mulheres; IMC: índice de massa corporal; UTI: unidade de terapia intensiva.

**Tabela 2 t6:** Sintomas típicos dos pacientes com COVID-19 dos nove estudos retrospectivos

Sintomas	Jin et al.^(^^18^^)^			Lin et al.^(^^19^^)^	Pan et al.^(^^20^^)^			Wan et al.^(^^21^^)^	Redd et al.^(^^22^^)^			
	Com sintomas GI	Sem sintomas GI	Valor de p	Total	Total	Com sintomas GI	Sem sintomas GI	Total	Total	Com sintomas GI	Sem sintomas GI	Valor de p
Pacientes	74	577		95	204	103	101	230	318	195	123	
Febre	63/74 (85,14) >38,5°C: 29/74 (39,19)	482/577 (83,54) >38,5°C: 101/577 (17,50)	0,867 >38,5°C: <0,001	Sim		95/103 (92,23)		193/230 (84)	258/318 (81,3)	161/195 (82,6)	97/123 (78,9)	0,41
Fadiga	23/74 (31,08)	96/577 (16,64)	0,004			54/103 (52,42)		43 (19)	183/318 (57,5)	127/195 (65,1)	56/123 (45,5)	0,0006
Mialgia	10/74 (13,51)	61/577 (10,57)	0,430			15/103 (14,56)			123/318 (38,7)	96/195 (49,2)	27/123 (22,0)	<0,0001
Calafrios									72/318 (22,6)	50/195 (25,6)	22/123 (17,9)	0,11
Diaforese									15/318 (4,7)	12/195 (6,2)	3/123 (2,4)	0,13
Artralgia									8/318 (2,5)	4/195 (2,1)	4/123 (3.3)	0,51
Tosse seca	53/74 (71,62)	382/577 (66,20)	0,431					159/230 (69)	247/318 (77,7)	156/195 (80,0)	91/123 (74,0)	0,21
Tosse produtiva	29/74 (39,19)	198/577 (34,32)	0,438					98/230 (43)	45/318 (14,2)	33/195 (16,9)	12/123 (9,8)	0,74
Dispneia	8/74 (10,81)	19/577 (3,30)	0,007					30 (13)	191/318 (60,1)	107/195 (54,9)	84/123 (68,3)	0,02
Dor de garganta	6/74 (8,11)	93/577 (16,12)	0,085						54/318 (17,0)	42/195 (21,5)	12/123 (9,8)	0,0064
Rinorreia									36/318 (11,4)	26/195 (13,4)	10/123 (8,1)	0,15
Obstrução nasal	2/74 (2,70)	35/577 (6,07)	0,419									
Tontura												
Anosmia									32/318 (10,1)	26/195 (13,3)	6/123 (4,9)	0,0146
Ageusia									24/318 (7,6)	21/195 (10,9)	3/123 (2,4)	0,0057
Cefaleia	16/74 (21,62)	51/577 (8,84)	0,002					19 (8)				
Hemoptise	3/74 (4,05)	8/577 (1,39)	0,119					3 (1)				
**Sintomas**	**Nobel et al.**^(^^23^^)^	**Cholankeril et al.**^(^^24^^)^	**Zhang et al.**^(^^25^^)^	**Zhou et al.**^(^^26^^)^								
	**Total**	**Total**	**Total**	**Total**	**Total equipe médica**	**Com sintomas GI equipe médica**	**Sem sintomas GI equipe médica**	**Valor de p**	**Total não médicos**	**Com sintomas GI não médicos**	**Sem sintomas GI não médicos**	**Valor de p**
Pacientes	278	116	140	254	93	23	70		161	43	118	
Febre		76,70	110/120 (91,7)	213/254 (83,9)	80/93 (86)	19/23 (83)	61/70 (87)	0,729	133/161 (82)	39/43 (91)	94/118 (80)	0,157
Fadiga			90/120 (75,0)	133/254 (52,4)	52/93 (56,)	12/23 (52)	40/70 (57)	0,809	81/161 (50)	29/43 (67)	52/118 (44)	0,012
Mialgia		52,50		86/254 (33,9)	41/93 (44,)	10/23 (44)	31/70 (44)	1	45/161 (28)	17/43 (40)	28/118 (24)	0.073
Calafrios												
Diaforese												
Artralgia												
Tosse seca		94,8	90/120 (75,0)	98/254 (38,6)	41/93 (44)	7/23 (30)	34/70 (49)	0,152	57/161 (35)	14/43 (33)	43/118 (36)	0,712
Tosse produtiva				107/254 (42,1)	31/93 (33)	6/23 (26)	25/70 (36)	0,454	76/161 (47)	17/43 (40)	59/118 (50)	0,286
Dispneia		58	44/120 (36,7)	10/254 (3,9)	2/93 (2,1)	1/23 (4)	1/70 (1)	0,435	8/161 (5,0)	2/43 (5)	6/118 (5)	1,00
Dor de garganta				16/254 (6,3)	6/93 (6,4)	0/23 (0)	6/70 (9)	0,33	10/161 (6,2)	6/43 (14)	4/118 (3)	0,023
Rinorreia												
Obstrução nasal												
Tontura				18/254 (7,1)	10/93 (11)	4/23 (17)	6/70 (9)	0,256	8/161 (5,0)	5/43 (12)	3/118 (3)	0,032
Anosmia												
Ageusia												
Cefaleia												
Hemoptise												

Resultados expressos como n, média±desvio padrão, n/n total (%).

GI: gastrintestinal.

**Tabela 3:** Sintomas gastrintestinais dos pacientes com COVID-19 dos nove estudos retrospectivos

	Jin et al.^(^^18^^)^	Lin et al.^(^^19^^)^			Pan L et al.^(^^20^^)^	Wan et al.^(^^21^^)^	Redd et al.^(^^22^^)^	Nobel et al.^(^^23^^)^	Cholankeril et al.^(^^24^^)^	Zhang et al.^(^^25^^)^	Zhou et al.^(^^26^^)^		
		Total	Admissão	Durante hospitalização							Total	Com sintomas GI equipe médica	Com sintomas GI não médicos
Pacientes	651	95			204	230	318	278	116	140	254	23	43
Sintomas TGI	74/651 (11,4)	58/95 (61,1)	11/95 (11,6)	47/95 (49,5)	103/204 (50,5)	49/230 (21)	195/318 (61,3)	97/278 (35)	37 (31,9)	55/139 (39,6)	66/254 (26,0)	23/23 (100)	43/43 (100)
Diarreia	53/651 (8,14)	23/95 (24,2)	5/95 (5,3)	18/95 (18,9)	35/103 (33,98)	49/230 (21)	107/318 (33,7)	56/278 (20)	12 (10,3)	18/139 (12,9)	46/254 (18,1)	19/23 (86,6)	27/43 (62,8)
N/V	N: 10/651 (1,53)	N: 17/95 (17,9)	N: 3/95 (3,2)	N: 14/95 (14,7)	V: 4/103 (3,88)		N: 84/318 (26,4)	63/278 (23)	12 (10,3)	N: 24/139 (17,3)	N: 21/254 (8,3)	N: 5/23 (21,7)	N: 16/43 (37,2)
	V: 11/651 (1,69)	V: 4/95 (4,2)	V: 0/95 (0)	V: 4/95 (4,2)			V: 49/318 (15,4)			V: 7/139 (5,0)	V: 15/254 (5,9)	V: 1/23 (4,3)	V: 14/43 (32,6)
Anorexia		17/95 (17,9)	5/95 (5,3)	12/95 (12,6)	81/103 (78,64)		110/318 (34,8)		22 (25,3)	17/139 (12,1)			
Dor abdominal		2/95 (2,1)	0/95 (0)	2/95 (2,1)	2/103 (1,94)	3/230 (1)	46/318 (14,5)		10 (8,8)	8/139 (5,8)	3/254 (1,2)	0/23 (0)	3/43 (7,0)
Perda de peso							30/318 (9,4)						
Melena						10/230 (4)	2/318 (0,63)						
Refluxo		2/95 (2,1)	1/95 (1,1)	1/95 (1,1)			2/318 (0,63)						
Disfagia							1/318 (0,31)						
Odinofagia							1/318 (0,31)						
Hematoquezia/HDA		2/95 (2,1)	0/95 (0)	2/95 (2,1)			1/318 (0,31)						
Constipação							3/318 (0,94)						

Resultados expressos como n ou n/n total (%).

GI: gastrintestinal; TGI: trato gastrintestinal; N: náusea; V: vômito; HDA: hemorragia digestiva alta.

**Tabela 4:** Sintomas típicos e gastrintestinais dos pacientes com COVID-19 das metanálises

	Sultan et al.^(^^8^^)^	Cheung et al.^(^^27^^)^ – metanálise	Cheung et al.^(^^27^^)^ – coorte de Hong Kong	Cao et al.^(^^28^^)^	Li et al.^(^^29^^)^
Pacientes	10.890	4.243	59	46.959	1.994
Idade		45,1 (IIQ: 41,0-54,8)	58,5 (IIQ: 43,5-68,0; range: 22-96)	46,62 (IC95%: 31,710-61,531)	
Sexo		H: 57,3	H: 27/59 (45,8)	H: 55,6 (IC95%: 0,530-0,602)	H: 60 (IC95%: 0,54-0,65)
Comorbidades				35,6 (IC95%: 0,267-0,444)	
Sintomas GI		Total: 17,6 (IC95%: 12,3-24,5)	15/59 (25,4)	6,8 (IC95%: 0,044-0,092)	
		60 estudos e 4.243 pacientes			
		China: 16,1 (IC95%: 10,9-23,0)			
		53 estudos e 4.198 pacientes			
		Outros países: 33,4 (IC95%: 15,2-58,3)			
		7 estudos e 45 pacientes			
Anorexia		26,8 (IC95%: 16,2-40,8) 18 estudos			
Náusea/vômito	Total: 7,8 (7,1-8,5)	10,2 (IC95%: 6,6-15,3)	1/59 (1,7)		3,90
	26 estudos e 5.955 pacientes	32 estudos			
	China: 5,2 (4,4-5,9)				
	19 estudos e 4.054 pacientes				
	Outros países: 14,9 (13,3-16,6)				
	7 estudos e 1.901 pacientes				
Diarreia	Total: 7,7 (7,2-8,2)	12,5 (IC95%: 9,6-16,0)	13/59 (22,0)	6,8 (IC95%: 0,044-0,092)	4,80
	43 estudos e 10.676 pacientes				
	China: 5,8 (5,3-6,4)				
	32 estudos e 8.612 pacientes				
	Outros países: 18,3 (16,6-20,1)				
	11 estudos e 2.064 pacientes				
Dor abdominal	Total: 3,6 (3,0-4,3)	9,2 (IC95%: 5,7-14,5)	7/59 (11,9)		
	15 estudos e 4.031 pacientes	12 estudos			
	China: 2,7 (2,0-3,4)				
	10 estudos e 2.447 pacientes				
	Outros países: 5,3 (4,2-6,6)				
	5 estudos e 1.584 pacientes				
PCR fezes	15/50 (30)	48,1% (IC95%: 38,33-57,94)	9/59 (15,3)		
	7/10 (70)	12 estudos e 138 pacientes			
	44/153 (29)				
Febre			56/59 (94,9)	87,3 (IC95%: 0,838-0,909)	88,50
Tosse seca			22/59 (37,3)	58,1 (IC95%: 0,502-0,660)	68,60
Dor de garganta				12,0 (IC95%: 0,062-0,177)	
Tosse produtiva				29,4 (IC95%: 0,171-0,417)	28,20
Dor no peito				31,2 (IC95%: −0,024-0,648)	
Mialgia ou fadiga				35,5 (IC95%: 0,253-0,456)	35,80
Cefaleia ou tontura				9,4 (IC95%: 0,063-0,126)	12,20
Dispneia			4/59 (6,8)	38,2 (IC95%: 0,246-0,520)	21,90

IIQ: intervalo interquartil; IC: intervalo de confiança; H: homens; GI: gastrintestinal.

Jin et al., fizeram um estudo retrospectivo na China que analisou 651 pacientes (331 homens e 320 mulheres) confirmados com COVID-19 na província de Zhejiang, com média de idade de 46,14±14,19 anos. Destes, 74 (11,4%) apresentaram pelo menos um sintoma gastrintestinal como náusea, vômito e diarreia na admissão hospitalar. O sintoma gastrintestinal mais comum foi a diarreia (8,14%), com duração média de 4 dias, e a maioria dos casos foi autolimitada. Dos pacientes com sintomas gastrintestinais, 29 tiveram significativamente maiores taxas de febre >38,5°C (39,19% *versus* 17,50% dos sem sintomas gastrintestinais; p<0,001), 23 fadiga (31,08% *versus* 16,64%; p=0,004), oito dispneia (10,81% *versus* 3,30%; p=0,007) e 16 cefaleia (21,62% *versus* 8,84%; p=0,002). Também houve diferença significativa entre os pacientes com doença hepática crônica (10,81% com sintomas gastrintestinais *versus* 2,95% sem sintomas gastrintestinais; p=0,004) e gravidade da doença na admissão, com falência respiratória, choque e falência múltipla de órgãos com necessidade de ventilação mecânica e internação na unidade de terapia intensiva (UTI; 22,97% *versus* 8,14%; p<0,001). Em relação aos achados de exames, os níveis séricos de sódio foram menores nos pacientes com sintomas gastrintestinais (137,65mmol/L *versus* 138,33mmol/L; p=0,016), tendendo a ter doença mais grave, e a aspartato aminotransferase (AST) teve taxa maior nesses pacientes (29,35U/L *versus* 24,4U/L; p=0,02). Pacientes com sintomas gastrintestinais tiveram mais complicações significativas durante o tratamento, como evolução para SARS com necessidade de internação em UTI (6,67% *versus* 2,08%; p=0,034) e lesão hepática (17,57% *versus* 8,84%; p=0,035), do que os sem sintomas gastrintestinais. A presença do RNA do vírus nas fezes foi detectada em poucos pacientes, apenas em três de nove pacientes com sintomas gastrintestinais. Pela pequena amostra, não avaliaram as implicações da transmissão oral-fecal, precisando de mais investigação. Entretanto, vale lembrar que esse estudo possui algumas limitações, pois não era de coorte e não identificou os pacientes com sintomas gastrintestinais que não tinham os sintomas típicos de tosse e febre.^(^^18^^)^

No estudo de Lin et al., com 95 pacientes (50 mulheres e 45 homens) de Zhuhai, China, com COVID-19 e média de idade 45,3±18,3 anos, 58 (61,0%) apresentaram sintomas gastrintestinais, sendo que 11 (11,6%) foram na admissão hospitalar e 47 (49,5%) durante a internação. Esses últimos provavelmente tiveram o quadro agravado pelo uso de medicamentos como antibióticos. Os principais sintomas gastrintestinais foram diarreia, anorexia e náusea, em 24,2%, 17,9% e 17,9%, respectivamente. A presença do RNA do vírus nas fezes foi testada em 65 pacientes (42 com e 23 sem sintomas gastrintestinais), sendo positiva em 22 (52,4%) dos que tinham sintomas gastrintestinais, e nove (39,1%) dos que não tinham. A presença do RNA do vírus nas fezes não indica necessariamente sintomas gastrintestinais mais severos, pois não mostrou diferença significativa entre os dois grupos (p=0,31). Entretanto, a presença do vírus no tecido indicou doença mais grave. Ao todo, seis pacientes foram submetidos à endoscopia (dois severos e quatro não severos), e um apresentou sangramento no esôfago com úlceras e erosões. Os dois pacientes severos também foram submetidos à proctoscopia, e o RNA do vírus foi detectado no esôfago, no estômago, no duodeno e no reto desses pacientes. Dos pacientes não severos, apenas um teve a presença do RNA viral no duodeno. O estudo conclui que o TGI pode ser uma potencial rota de transmissão e órgão-alvo para o SARS-CoV-2, e os sintomas gastrintestinais não podem ser subestimados.^(^^19^^)^

De acordo com o estudo descritivo, transversal, multicêntrico (três hospitais de Hubei, na China) de Pan et al., com 204 pacientes, sendo 107 homens, com média de idade de 52,91±15,98 anos, 103 (50,5%) relataram algum sintoma gastrintestinal, como falta de apetite (81; 78,6%), diarreia (35; 34,0%), vômitos (4; 3,9%) e dor abdominal (2; 1,9%). Se eliminarmos a falta de apetite, foram 38 (18,6%) pacientes com sintomas gastrintestinais. Seis pacientes (3%) relataram apenas sintomas digestivos. Foi observado que, com o aumento da severidade da doença, os sintomas gastrintestinais se tornaram mais pronunciados. Pacientes com sintomas gastrintestinais tiveram mais anormalidades laboratoriais, como elevação de alanina aminotransferase (ALT; 42,24U/L *versus* 29,53U/L; p=0,011) e AST (35,12U/L *versus* 27,48U/L; p=0,032), diminuição na contagem de monócitos (0,39x10^9^/L *versus* 0,46x10^9^/L; p=0,021), aumento do tempo de protrombina (13,13 segundos *versus* 12,53 segundos; p=0,024) e receberam mais antimicrobianos durante o tratamento (76,70% *versus* 61,39%; p=0,018), quando comparados com os sem sintomas gastrintestinais. Desse modo, esses pacientes tiveram mais chance de sofrer lesão hepática. Porém, algumas limitações devem ser consideradas, já que este foi um estudo retrospectivo, com amostra pequena e não testou a presença do SARS-CoV-2 nas fezes dos pacientes.^(^^20^^)^

Wan et al., fizeram um estudo retrospectivo multicêntrico com 14 hospitais na China, e mostraram que os sintomas gastrintestinais são comuns em paciente com COVID-19. De 230 pacientes com a doença, 129 eram homens, com média de idade 47,5 anos, e diarreia foi observada em 49 (21%) deles. Outros sintomas gastrintestinais, como dor abdominal (3,1%) e melena (10,4%), também foram relatados. Esses pacientes com diarreia tinham mais comorbidades (39% *versus* 22%; p=0,017), idade mais avançada (55 anos *versus* 46 anos; p=0,017) e apresentaram sintomas mais severos de problemas respiratórios, com necessidade de UTI (31% *versus* 11%; p=0,0015), ventilação mecânica (12% *versus* 2%; p=0,0036) e morte (8% *versus* 1%; p=0,020). O estudo não tem dados de pacientes assintomáticos, o que pode superestimar a prevalência dos sintomas gastrintestinais na COVID-19.^(^^21^^)^

Um estudo de coorte feito por Redd et al., em nove hospitais em Massachusetts, Estados Unidos, avaliou a presença de sintomas gastrintestinais em 318 pacientes adultos (174 homens) com COVID-19 na admissão hospitalar e média de idade 63,4±16,6 anos. Apresentaram sintomas gastrintestinais na admissão 195 pacientes (61,3%), dentre os quais anorexia (110; 34,8%), diarreia (107; 33,7%), náusea (84; 26,4%) e vômito (49; 15,4%). Os sintomas gerais que foram associados significativamente com os gastrintestinais em relação aos pacientes sem sintomas gastrintestinais, foram fadiga (65,1% *versus* 45,5%; p=0,0006), mialgia (49,2% *versus* 22,0%; p<0,0001), dor de garganta (21,5% *versus* 9,8%; p=0,0064), anosmia (13,3% *versus* 4,9%; p=0,0146) e ageusia (10,9% *versus* 2,4%; p=0,0057). Um subgrupo de 202 pacientes precisou de hospitalização até a finalização do artigo em questão, sendo que 35 (17,5%) foram para a UTI, 26 (13,0%) precisaram de ventilação mecânica e 32 (15,8%) foram a óbito, mas não foi observada diferença significativa com piora do quadro clínico em pacientes com e sem sintomas gastrintestinais. As limitações do estudo foram ser um desenho retrospectivo, faltar instrumentos de validação de sintomas, focar apenas em dados hospitalares e o fato de terem sido excluídos pacientes ambulatoriais com doença menos severa. Além disso, o artigo não avaliou a presença do vírus nas fezes desses pacientes.^(^^22^^)^

Nobel et al., fizeram estudo retrospectivo de caso controle que comparou os sintomas de 278 pacientes positivos para SARS-CoV-2 com 238 pacientes negativos nos Estados Unidos. Os sintomas gastrintestinais considerados foram diarreia ou náusea/vômito. Eles encontraram diferença significativa entre a presença de sintomas gastrintestinais em pacientes com e sem COVID-19 (p=0,04). A presença desses sintomas foi associada a um risco maior de 70% de ter COVID-19. Entre os pacientes com COVID-19, (97; 35%) apresentaram sintomas gastrintestinais, sendo estes diarreia (56; 20%) e náusea/vômito (63; 23%). Pacientes com sintomas gastrintestinais tiveram duração maior da doença quando comparados com os sem esses sintomas (33% *versus* 22%; p=0,048). As limitações do estudo foram curto período de acompanhamento dos pacientes, estudo retrospectivo e a identificação dos sintomas gastrintestinais ter sido dependente de documentação bem feita. Esse artigo também não avaliou a presença do vírus nas fezes dos pacientes.^(^^23^^)^

Em um estudo de coorte de Cholankeril et al., em uma instituição dos Estados Unidos, os sintomas gastrintestinais foram relatados em 31,9% (97) dos pacientes com COVID-19, sendo os sintomas mais comuns perda de apetite (22; 25,3%), náusea/vômitos (12; 10,3%) e diarreia (12; 10,3%). Foram incluídos no estudo 116 pacientes com COVID-19, sendo a maioria do sexo masculino (53,4%) e com média de idade de 50 anos. Nenhum paciente apresentou sintomas gastrintestinais isolados como manifestação inicial. A duração média dos sintomas gastrintestinais foi de 1 dia, sendo significativamente menor que a duração dos sintomas respiratórios (p<0,001). Elevação de AST esteve correlacionada com a severidade da doença (p=0,009). Esse estudo apresentou limitações, como pacientes de apenas instituição (tendência regional), o fato de a documentação dos sintomas extrapulmonares poder estar incompleta e o diagnóstico de COVID-19 ter sido feito apenas em pacientes com sintomas respiratórios. O artigo não avaliou a presença do vírus nas fezes desses pacientes.^(^^24^^)^

Para Zhang et al., que estudaram em 140 pacientes da China com COVID-19, 39,6% deles apresentaram sintomas gastrintestinais. Nesse estudo, a proporção entre homens e mulheres foi praticamente a mesma, e a média de idade foi de 57,0 anos. Os principais sintomas foram febre (91,7%), tosse (75,0%) e fadiga (75,0%). Eles apontam que eosinopenia com ou sem linfopenia em pacientes com sintomatologia e alterações radiológicas pode ser um potencial indicador para diagnóstico da COVID-19. Além disso, encontraram que doenças alérgicas, asma e doença pulmonar obstrutiva crônica (DPOC) não são fatores de risco para infecção por SARS-CoV-2, mas estudos com amostras maiores são necessários para confirmar esses achados. Idade avançada (p<0,001), comorbidades (p=0,002) e anormalidades laboratoriais estão associadas com severidade da doença. Essas alterações laboratoriais são altos valores de dímero D (p<0,001), proteína C-reativa (p<0,001), procalcitonina (p<0,001) e leucopenia (p<0,014). Nesse estudo, pacientes com DPOC e fumantes apresentaram menor risco de serem infectados pelo SARS-CoV-2, mas a evolução do quadro nesses pacientes foi mais severa. Entretanto, a relação com fumantes com COVID-19 ainda é incerta. Fumantes e pacientes com DPOC são mais suscetíveis a infecções pelo MERS-CoV, pois o tabaco e a DPOC aumentam a expressão do transportador DPP4, que é o receptor para esse vírus.^(^^25^^)^

Zhou et al., avaliaram 254 pacientes de um centro de Wuhan com confirmação de COVID-19. Eles compararam a equipe médica infectada separadamente dos pacientes não médicos. Na amostra, 139 eram mulheres, a média de idade foi de 50,6 anos, e 161 não pertenciam à equipe médica. Os sintomas mais relatados em todos os pacientes foram febre (211; 83%), tosse (98; 38,6%) e sintomas gastrintestinais (66; 26%). Nos pacientes não médicos, a proporção de sintomas gastrintestinais em mulheres foi significativamente maior que nos homens (62,8% *versus* 37,2%; p=0,033). Além disso, manifestações clínicas, como dor de garganta (14% *versus* 3%; p=0,023), tontura (12% *versus* 3%; p=0,032), fadiga (67% *versus* 44%; p=0,012), hemoglobina baixa (116,7 *versus* 133; p=0,028), proteína C-reativa alta (7,3 *versus* 3,8; p=0,021) e ALT alta (64,1 *versus* 46,6; p=0,049), também foram significativamente mais frequentes nos pacientes não médicos com sintomas gastrintestinais. Entretanto, não houve correlação significativa entre os sintomas e as características clínicas dos pacientes da equipe médica com e sem sintomas gastrintestinais. Uma explicação do porquê a equipe médica foi menos susceptível aos sintomas gastrintestinais é que a grande parte desse grupo é formada por enfermeiros jovens sem comorbidades. Esse estudo possui limitações. A maioria dos casos incluídos foram confirmados clinicamente, portanto ele possui pacientes sem o teste de PCR para SARS-CoV-2. Além disso, muitos pacientes não foram acompanhados, pois ainda estavam hospitalizados no momento da submissão do artigo.^(^^26^^)^

Cipriano et al., fizeram uma revisão sistemática com 6 estudos com pacientes da China, a qual aponta para a possibilidade de infecção do SARS-CoV-2 pelo trato gastrintestinal e transmissão fecal-oral. No estudo, 53,42% das amostras de fezes testaram positivo para a presença do SARS-CoV-2 em 73 pacientes hospitalizados. Essas amostras se mantiveram positivas entre 1 e 12 dias, e 23,29% dos pacientes tiveram fezes positivas para o RNA do vírus após a negativação das amostras respiratórias. Os autores concluem que o *swab* anal pode ser tão importante quando o *swab* nasofaríngeo, mesmo em pacientes assintomáticos. Médicos devem considerar, antes da alta hospitalar, que a infecção gastrintestinal e a potencial transmissão fecal-oral podem permanecer até após o desaparecimento viral do trato respiratório.^(^^30^^)^

Cheung et al., fizeram estudo interessante sobre os sintomas gastrintestinais da COVID-19 e a presença de RNA viral nas fezes. Eles compararam uma metanálise com uma coorte de Hong Kong. Na coorte de 59 pacientes com a doença, 15 (25,4%) apresentaram sintomas gastrintestinais e 9 (15,3%) tiveram suas fezes testadas positivas para o RNA viral, sendo que 38,5% e 8,7% foram nos com e nos sem diarreia, respectivamente (p=0,019). Na metanálise, que incluiu 60 estudos e 4.243 pacientes, a prevalência de pacientes com sintomas gastrintestinais foi de 17,6%, sendo 16,1% em estudos da China e 33,4% em outros países (Coreia do Sul, Singapura, Vietnam, Estados Unidos e Reino Unido). Os sintomas gastrintestinais avaliados foram anorexia (26,8%), diarreia (12,5%), náusea/vômito (10,2%) e dor abdominal (9,2%). A prevalência do RNA do vírus nas fezes foi de 48,1%. Dessas amostras, 70,3% testaram positivo nas fezes, podendo persistir por mais de 33 dias do início dos sintomas, mesmo após o sistema respiratório ter resultado negativo para RNA viral. Essa metanálise também mostrou que a prevalência de doença severa foi mais comum em pacientes com sintomas gastrintestinais do que os sem (17,1% *versus* 11,8%). As limitações do estudo são subnotificação dos sintomas gastrintestinais e menor número de estudos fora da China. Com isso, os profissionais da saúde devem se atentar para coleta de fezes e em procedimentos como endoscopia em pacientes com COVID-19.^(^^27^^)^

A revisão sistemática e metanálise com 31 artigos e 46.959 pacientes da China, por Cao et al., descreveu a prevalência de sintomas como febre (87,3%), tosse seca (58,1%), dispneia (38,2%), mialgia ou fadiga (35,5%), dor no peito (31,2%), tosse produtiva (29,4%), dor de garganta (12%), cefaleia (9,4%) e diarreia (6,8%). A incidência de necessidade de UTI, SARS, falência múltipla de órgãos e mortalidade foi de 29,3%, 28,8%, 8,5% e 6,8%, respectivamente. As limitações desse estudos foram não haver casos estrangeiros e não comentar sobre a presença viral nas fezes.^(^^28^^)^

A metanálise feita por Li et al., com dez artigos e 1.994 pacientes da China, apresentou resultados parecidos. Os principais sintomas relatados foram febre (88,5%), tosse (68,6%), mialgia ou fadiga (35,8%), tosse produtiva (28,2%) e dispneia (21,9%). Outros sintomas foram cefaleia ou tontura (12,1%), diarreia (4,8%) e náusea e vômito (3,9%). Também foram registrados linfopenia (64,5%), aumento da proteína C-reativa (44,3%), aumento da desidrogenase láctica (28,3%) e leucopenia (29,4%). Além disso, os homens foram mais acometidos (60%), e a taxa de fatalidade foi de 5%. Esse estudo apresentou limitações pelo número e pela qualidade dos estudos incluídos.^(^^29^^)^

Sultan et al., fizeram revisão sistemática e metanálise com 47 estudos com 10.890 pacientes. A prevalência de diarreia foi de 7,7%, de náusea e vômito de 7,8% e da dor abdominal de 2,7%. Ao comparar os dados da China e de outros países, diarreia (5,8% *versus* 18,3%), náusea e vômito (5,2% *versus* 14,9%), dor abdominal (2,7% *versus* 5,3%) e anormalidades hepáticas foram mais prevalentes fora da China. As alterações do fígado estudadas foram elevação de AST (total: 15,0%; China: 14,9%; outros países: 20,0%), ALT (total: 15,0%; China: 14,9%; outros países 19,0%) e bilirrubina (total ou China: 16,7%). Eles não fizeram análise mais profunda sobre a presença do RNA viral nas fezes desses pacientes, mas comentam que encontraram prevalência variável de 15/50 (30%), 7/10 (70%) e 44/153 (29%). Os autores também disponibilizam um manual de recomendações de boas práticas no manejo da COVID-19 com sete itens, sendo os principais obter história detalhada dos sintomas (típicos e do TGI), verificar valores de função hepática na admissão de pacientes para monitoramento durante hospitalização e avaliar as reações adversas de medicamentos utilizados no tratamento. Sobre as limitações, os pacientes estavam hospitalizados (prevalência pode estar sub ou superestimada), os sintomas podem ter sido analisados de maneira inconsistente, faltaram informações de exames laboratoriais, não houve informação sobre sintomas gastrintestinais terem sido avaliados sistematicamente na admissão, entre outras. Eles concluem que a monitorização das enzimas hepáticas pode ser benéfica, e ainda não há evidências que suportam o teste de PCR viral das fezes como diagnóstico ou para monitoramento da COVID-19 como rotina da prática clínica, pois os resultados de outras metanálises são conflitantes e são necessários mais estudos sobre o tema.^(^^8^^)^

## DISCUSSÃO

Com a avaliação de todos esses estudos, percebe-se que ainda há muita variação nos resultados em vários pontos, e os quesitos estudados também foram muito diferentes.

A média de idade dos pacientes variou entre 45,1 e 58,5 anos, mas apenas um artigo correlacionou significativamente idade avançada com sintomas gastrintestinais.^(^^21^^)^ Em outro artigo, a idade avançada foi relacionada com gravidade da doença, mas sem avaliar se esses pacientes tinham sintomas gastrintestinais.^(^^25^^)^ De modo geral, todos os artigos tiveram mais homens acometidos com a COVID-19 do que mulheres, e apenas um encontrou diferença significativa entre mulheres não médicas e mais sintomas gastrintestinais.^(^^26^^)^ Esses resultados sugerem que idade avançada e sexo masculino são fatores de risco para a infecção. O gene do receptor ECA2, alvo do SARV-CoV-2, está localizado no cromossomo X. Um estudo, ainda em revisão, verificou que a expressão da ECA2 aumenta no sexo feminino e com hormônios sexuais (que diminuem com aumento da idade), e citocinas inflamatórias diminuem essa expressão (que aumentam com idade e doenças crônicas). Isso é contrário ao que é pensado atualmente, que aumento da ECA2 é culpado pelo pior prognóstico da COVID-19. SARS-CoV-2 se liga diretamente à ECA2, provocando *downregulation* (regulação negativa) desse receptor, que fica ainda em menor quantidade em homens, por exemplo, podendo explicar o pior prognóstico e a maior infecção neles.^(^^31^^)^

Em relação às comorbidades dos pacientes infectados, as mais citadas foram hipertensão arterial, *diabetes mellitus*, doença cerebrovascular, doença cardiovascular, doença pulmonar, doença renal, doença hepática, câncer, entre outras. Porém, apenas três artigos tiveram relação significativa entre sintomas gastrintestinais e comorbidades, sendo que um deles é a doença hepática crônica.^(^^18^^,^^21^^,^^25^^)^ Entretanto, a relação entre a COVID-19 e as comorbidades também se mostrou muito variada. Foram abordadas comorbidades muito frequentes na população e que podem ter significado importante na infecção pelo SARS-CoV-2 e prognóstico.

Os sintomas típicos mais descritos são comuns em infecções do trato respiratório, como febre (76,7% a 94,9%), tosse seca (38,6% a 77,7%), tosse com expectoração (14,2% a 43,0%), fadiga (16,6% a 75,0%), dispneia (3,3% a 60,1%), cefaleia (8,0% a 21,6%) e dor de garganta (8,11% a 17,0%).^(^^8^^,^^18^^-^^29^^)^ Outros sintomas, como anosmia e ageusia, estão sendo muito relacionados com a COVID-19, mas apenas um estudo os verificou (10,1% e 7,6%, respectivamente). Esse estudo encontrou correlação entre presença de fadiga, mialgia, dispneia, dor de garganta, anosmia e ageusia com sintomas gastrintestinais.^(^^22^^)^ Outros dois estudos também identificaram relações entre sintomas gastrintestinais e sintomas típicos, sendo que um foi com febre acima de 38,5°C, fadiga, dispneia e cefaleia e o outro com fadiga, dor de garganta e tontura.^(^^18^^,^^26^^)^

Sobre os sintomas gastrintestinais, a prevalência teve grande variação, ficando entre 6,8% e 61,3% nesses estudos. Isso mostra que pode haver uma tendência regional neles, e estudos com muitos pacientes e de diversos países são necessários para melhorar essa relação entre o TGI e a COVID-19. Importante lembrar que duas metanálises fizeram esse estudo entre China e outros países e perceberam que os demais locais apresentaram maiores taxas de sintomas gastrintestinais.^(^^8^^,^^27^^)^ Entretanto, ainda eram poucos os outros países e muitos eram na própria Ásia. De qualquer forma, isso mostra que os sintomas gastrintestinais são importantes nessa doença, podendo ter grande impacto em alguns países, e que deve ser avaliado em anamnese de pacientes com suspeita. Os sintomas gastrintestinais mais citados foram diarreia (8,14% a 33,7%), náusea/vômito (1,53% a 26,4%), anorexia (12,1% a 40,0%) e dor abdominal (0% a 14,5%).^(^^8^^,^^18^^-^^29^^)^

Diversos estudos apontam relações entre alterações laboratoriais e sintomas gastrintestinais ou severidade da doença. Lesão hepática (elevação de AST, ALT e bilirrubina) foi a mais avaliada, mas também foram verificadas elevação de proteína C-reativa, procalcitonina, desidrogenase láctea, dímero e protrombina, além de diminuição de sódio sérico, leucócitos, linfócitos e monócitos.^(^^8^^,^^18^^,^^20^^,^^24^^,^^25^^,^^29^^)^ Com isso, é muito importante a avaliação desses parâmetros em pacientes internados com COVID-19, pois eles podem indicar prognóstico pior da doença.

Um importante fator que foi pouco avaliado entre os estudos foi a presença do RNA viral em amostras de fezes de pacientes infectados com SARS-CoV-2. Esse dado nos ajudaria a entender se existe a possibilidade de transmissão fecal-oral nessa doença. Apenas quatro estudos fizeram essa análise, sendo duas metanálises e dois retrospectivos. Esse teste foi positivo entre 0% e 48,1% dos casos, com grande heterogeneidade nos resultados, mas os pacientes com sintomas gastrintestinais apresentaram mais frequentemente a presença do RNA do vírus nas fezes.^(^^8^^,^^18^^,^^19^^,^^27^^)^ Alguns também identificaram que esse resultado se manteve positivo mesmo após a negativação de amostras do trato respiratório. Em um deles, 53,42% das amostras de fezes foram positivas para o vírus, e 23,29% ainda estavam positivas quando o trato respiratório negativou.^(^^30^^)^ Em outro, 70,3% dos pacientes que tiveram fezes positivas para o vírus (48,1%) também se mantiveram positivas após o *clearence* (clareamento) respiratório.^(^^27^^)^ Entretanto, os estudos não avaliaram se o RNA presente nas fezes representam vírus infectante ou apenas resíduos virais, sem capacidade de infectar. Esses dados nos levam à dúvida se o teste de PCR nas fezes de pacientes com COVID-19 deve ser feito rotineiramente ou não. Assim, mais estudos devem se preocupar e avaliar essa questão, visto que a possível transmissão fecal-oral pode ter impacto grande em países em desenvolvimento que possuem falta de saneamento básico em muitos locais.

Por fim, a necessidade de admissão em UTI variou entre 2,08% a 31,0% e casos que evoluíram a óbito entre 0% a 17,65%. Vários estudos comentaram sobre isso, mas apenas dois apresentaram diferença significativa entre a presença de sintomas gastrintestinais e a necessidade de UTI ou morte.^(^^18^^,^^21^^)^ Assim, torna-se muito importante a investigação desses pacientes com sintomas gastrintestinais desde o início do quadro infeccioso, visto que, uma parcela deles, maior do que os sem esses sintomas, pode desenvolver doença mais grave e necessitar de UTI, ou até evoluir a óbito.

Os artigos dessa revisão possuem diversas limitações. De forma geral, a avaliação dos sintomas foi feita de maneira diferente entre eles, pode haver subnotificação dos sintomas gastrintestinais, poucos foram feitos com pacientes fora da China, os desenhos foram retrospectivos e com pequenas amostras, muitos não avaliaram a presença de RNA viral nas fezes, não incluíram pacientes assintomáticos, os dados eram hospitalares e o acompanhamento dos pacientes foi curto. Além disso, por essa revisão incluir diferentes desenhos de estudos, há o risco de que as metanálises e revisões sistemáticas também contenham algum artigo retrospectivo, podendo ter resultados duplicados. Os variados desenhos de estudo, por si só, também são uma limitação dessa revisão, visto que o ideal seria utilizar um mesmo modelo.

## COMENTÁRIOS

Os altos índices de infecção pelo SARS-CoV-2 no mundo inteiro fizeram com que a Organização Mundial da Saúde decretasse estado de pandemia em 2020. Com isso, informações acerca dessa nova doença são de extrema importância, tanto de fisiopatologia, quanto de prevalência, severidade, fatores de risco, diagnóstico e tratamento. Com este trabalho, percebemos que o trato gastrintestinal pode ser muito influenciado pela doença, causando sintomas específicos (diarreia, dor abdominal, náusea e vômito) e alterações laboratoriais (principalmente de enzimas hepáticas). Os profissionais da saúde devem se atentar a isso, mantendo-se alerta a essas alterações, que podem ajudar no diagnóstico e a iniciar o tratamento precoce, para evitar mal prognóstico. Entretanto, os resultados de prevalência dos sintomas gastrintestinais foram muito diferentes, apresentando variação de 6,8% a 61,3%, sendo esses sintomas diarreia (8,14% a 33,7%), náusea/vômito (1,53% a 26,4%), anorexia (12,1% a 40,0%) e dor abdominal (0% a 14,5%). Essa importante variação demanda estudos maiores, com mais pacientes e de diversos países. Em relação à possibilidade de transmissão fecal-oral, a presença do RNA viral foi pouco testada, mas foi positiva entre 0% a 48,1%. Esses dados também variaram muito, impedindo que houvesse conclusão quanto a eles, mas também devem ser mais estudados, para que medidas de prevenção possam ser tomadas. O RNA do vírus nas fezes não indica, necessariamente, que haja transmissão fecal-oral, sendo importante sua avaliação em novos estudos para saber se essa presença é infectante ou não.
